# Resolution-Adaptive Binning Enhances Machine Learning Modeling by Interbatch and Multiplatform Orbitrap-Based Shotgun Mass Spectrometry Data Integration

**DOI:** 10.1021/acs.analchem.5c05874

**Published:** 2025-11-25

**Authors:** Hiu-Lok Ngan, Jialing Zhang, Kenneth Kin-Leung Kwan, Jacinth Wing-Sum Cheu, Li Zhong, Yike Guo, Xian Yang, Carmen Chak-Lui Wong, Hong Yan, Zongwei Cai

**Affiliations:** † State Key Laboratory of Environmental and Biological Analysis, Department of Chemistry, 26679 Hong Kong Baptist University, Hong Kong 999077, P. R. China; ‡ State Key Laboratory of Liver Research, Department of Pathology, Li Ka Shing Faculty of Medicine, The University of Hong Kong, Hong Kong 999077, P. R. China; § Centre for Oncology and Immunology, Hong Kong Science Park, Hong Kong 999077, P. R. China; ∥ Department of Computer Science, Hong Kong Baptist University, Hong Kong 999077, P. R. China; ⊥ Department of Computer Science and Engineering, Hong Kong University of Science and Technology, Hong Kong 999077, P. R. China; # Alliance Manchester Business School, The University of Manchester, Manchester M15 6PB, United Kingdom; ∇ Department of Biology, Hong Kong Baptist University, Hong Kong 999077, P. R. China; ○ Eastern Institute of Technology, Ningbo 315200, P. R. China

## Abstract

Machine learning (ML) modeling on mass spectrometry (MS)-based shotgun data facilitates feature selection and disease modeling. However, batch-specific models often struggle with limited transferability and generalizability, necessitating data integration from multiple batches and platforms. Traditional binning methods can either disintegrate or aggregate *m*/*z* features, making data combination unreliable. In this study, we introduce a mass resolution-adaptive binning and integration strategy to overcome these challenges. This approach recovers 88–99% of ground truth features in a low mass region (70–434 *m*/*z*) from 49 mixed standard solutions at 250, 500, and 1000 ppb. Compared to conventional methods, it demonstrates stable binning and integration across low (100–450 *m*/*z*), mid (450–900 *m*/*z*), and high (900–1500 *m*/*z*) mass regions, resulting in superior predictive models. Using a mouse model of hepatocellular carcinoma as a proof-of-concept study, we identify 10 generic metabolites that showcase advancements in using ambient MS imaging (MSI) data for modeling and deploy the attained model to shotgun data. This facilitates disease detection via various sample introduction methods, including MSI on liver cryosections (*F*1 score = 0.87) and glass smears (*F*1 score = 0.80), as well as rapid direct infusion analysis (recall = 0.89 and precision = 0.63). This novel mass resolution-adaptive binning and integration strategy offers a promising approach for integrating different data sets, potentially improving disease detection accuracy in MS applications.

## Graphical abstract

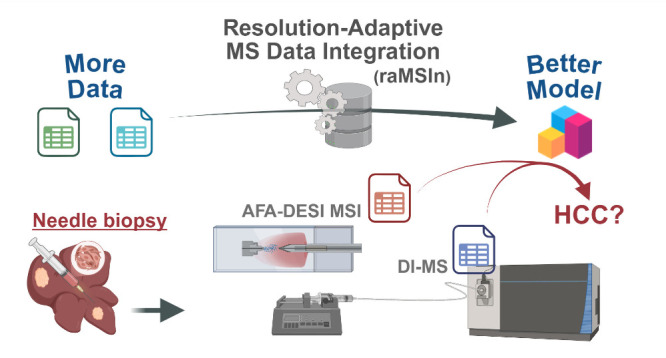

## Introduction

Cancer is the leading cause of death worldwide, with nearly 10 million deaths in 2020.^1^ Liver cancer ranked seventh in incidence but was the third most common cause of cancer-related deaths,^2^ having the highest mortality-to-incidence ratio among all cancer types at nearly 1:1.^2^ Early detection significantly improves outcomes, yet small tumor spots are difficult to palpate during physical examinations.^3^ When imaging tests like computed tomography or magnetic resonance imaging are inconclusive,^4^ fine-needle aspiration (FNA) is used for malignancy confirmation.^5,6^ This requires precise needle placement and adequate diagnostic cell collection.^7,8^ Needle biopsies are subsequently smeared for qualitative phenotyping examination by pathologists, which can lead to subjective and potentially inaccurate interpretations.^9^ Thus, a more robust and objective method for liver cancer detection is crucial.

Machine learning (ML) modeling on mass spectrometry (MS) data has become increasingly valuable for analyzing complex biological samples. High-resolution MS (HRMS)-based untargeted metabolomics is a high-throughput technique that profiles metabolites under 1500 Da and is gaining traction for its potential to uncover biomarkers linked to various diseases.^10^ Incorporating ML with metabolomics enhances the ability to identify patterns and features that may indicate disease states, including hepatocellular carcinoma (HCC).^11–17^

Separation methods like liquid chromatography (LC) coupled with MS^12,18,19^ provide broader metabolite coverage compared to shotgun analysis.^20–23^ However, ML modeling on LC/MS data faces challenges due to variability in tissue sampling and the sufficiency of data points, leading to poor transferability across data sets. Ambient Orbitrap-based MS imaging (MSI) offers a solution, allowing for multichannel untargeted metabolite profiling for a whole tissue section without the need for sample pretreatment. This technology generates extensive multipixel shotgun data without the interference of matrix ions, which can be advantageous for modeling.^21^

Despite these advancements, data integration in MS can be complicated by mass shifts in *m*/*z* values.^24^ Effective *m*/*z* binning is crucial for data preprocessing and scalable model development,^25^ as improper binning can introduce errors that propagate through data integration and downstream ML analyses. Evaluating different *m*/*z* binning approaches is essential for ensuring efficacious data integration and reliable ML modeling.

In this study, we propose a mass resolution-adaptive binning approach for MS data integration. We illustrate the dependency of mass resolution on *m*/*z* values and compare the quality of *m*/*z* features produced by the resolution-adaptive method with traditional constant and dynamic binning approaches. By assessing error propagation during data integration, we evaluate the biological insights and predictive power of HCC models driven by different binning strategies. The resolution-adaptive approach demonstrates transferability across MSI of glass smears and versatility for direct infusion MS (DI-MS). This study highlights its potential to integrate shotgun MS data across various sample introduction methods, batches, and MS platforms, thereby enhancing the robustness of ML modeling in metabolomics.

## Materials and Methods

### Chemical and Reagents

Acetonitrile (ACN), dimethylformamide (DMF), and xylene were purchased from RCI Labscan (Bangkok, Thailand). Methanol (MeOH) was obtained from Duksan (Ansan-si, Gyeonggi-do, Korea). Both DMF and MeOH were of HPLC grade, while ACN was of LC-MS grade. Absolute ethanol (EtOH), isopropanol (IPA), and 28% ammonia solution were sourced from VWR Chemicals BDH (Fontenay-sous-Bois, France). 2-Amino-6-chloropurine riboside, 2′-deoxyadenosine, 2′-deoxycytidine, 2′-deoxyguanosine, 2′-deoxyinosine, 3,5-diiodo-l-tyrosine, 3-iodo-l-tyrosine, 8-bromoguanosine, adenine, adenosine, ammonium formate powder, betaine, cytidine, dansyl-dl-norleucine, dansyl-dl-norvaline, dansyl-dl-phenylalanine, dansyl-dl-valine, dansyl-dl-α-amino-*n*-butyric acid, dl-4-chloro-phenylalanine, guanine, guanosine, hematoxylin and eosin Y (H&E), hypoxanthine, inosine, l-alanine, l-arginine, l-asparagine, l-aspartic acid, l-carnitine, l-glutamic acid, l-glutamine, l-glutathione, l-histidine, l-homocysteine, l-isoleucine, l-leucine, l-lysine, l-methionine, l-ornithine, l-phenylalanine, l-proline, l-serine, l-threonine, l-tryptophan, l-tyrosine, *N*-acetyl-dl-tryptophan, *N*_α_-acetyl-l-lysine, *N*_α_-dansyl-dl-tryptophan, *N*_ε_-acetyl-l-lysine, *S*-(5′-adenosyl)-l-homocysteine, and γ-amino butyric acid were purchased from Sigma-Aldrich (St. Louis, MO, USA). Water was purified by using a Milli-Q water system (Millipore, Billerica, MA, USA).

### Preparation of Hepatocellular Carcinoma Mouse Liver Tissues

Mouse livers were prepared for MSI and DI-MS. All animal experiments and study protocols were approved by the University of Hong Kong (HKU)’s Committee on the Use of Live Animals in Teaching and Research (CULATR), in accordance with the Animals (Control of Experiments) Ordinance of Hong Kong. Mice were sacrificed if body weight loss exceeded 20%, and the maximal tumor burden allowed was not exceeded. C57BL/6J mice from the Chinese University of Hong Kong were acclimatized for 1 week at 22 ± 2 °C and 60 ± 5% relative humidity under a 12 h light/dark cycle. A diethyl nitrosamine-tetrachloromethane (DEN-CCl_4_) chemical-induced HCC mouse model was established.^26^ Liver tissues from 12 HCC mice and 12 age-matched controls (AMCs) were snap-frozen, transferred onto dry ice, and stored at −80 °C before use. The harvested liver tissues were sliced into 10 μm thick cryosections using a cryostat (CryoStar NX70, Thermo Scientific, USA) and stored at −80 °C until analysis.

### Preparation of Glass Smears and Liver Extracts

FNA biopsies from the right liver were collected for glass smears and liver extracts with some modifications to existing protocols.^27,28^ A 21-gauge needle attached to a disposable 20 mL luer lock syringe was used to aspirate tissue while applying vacuum pressure for 10–20 s. For glass smears, 3 HCC/AMC pairs (6 total) of needle aspirates were spread on individual glass slides to form oval smears and stored at −80 °C until analysis. For liver extracts, 5 HCC/AMC pairs (10 total) were collected in individual 1.5 mL centrifuge tubes with 600 μL of ice-chilled ACN/DMF (1:1, v/v) and 3 stainless steel beads. After homogenization and centrifugation at 12,000*g* for 15 min at 25 °C, each 400 μL of supernatant was dried with an IR concentrator (NB-504CIR; N-Biotek MAX-UP, Korea), reconstituted in 200 μL of ACN/IPA/H_2_O (65:30:5, v/v/v), and diluted 10 times for electrospray ionization MS (ESI-MS) through DI. The reminding extracts were pooled for structural identification of biomarkers by ESI tandem MS (ESI-MS/MS, see the Supporting Information).

### Desorption Electrospray Ionization MS Imaging

Two independent MSI data sets from cryosections of left livers were collected on different MS platforms for data integration and model training (Figure 1a). Glass smear MSI data were used for validation (Figure 1b). An airflow-assisted desorption electrospray ionization (DESI) imaging platform (AFA-DESI, Beijing Victor, Beijing, China) was integrated with either a Hybrid Q-Exactive Orbitrap mass spectrometer (QE, Thermo Fisher Scientific, San Jose, CA, USA) or an Orbitrap Exploris 120 mass spectrometer (OE120, Thermo Fisher Scientific, San Jose, CA, USA). Six HCC/AMC pairs of liver sections and 3 pairs of glass smears were imaged on the OE120, while 4 pairs were analyzed on the QE. Frozen samples were dried at room temperature for 10 min prior to DESI-MSI. Data were acquired in negative ion full-scan mode across the *m*/*z* range of 100–1500 in centroid mode, with resolutions of 70,000 for QE and 60,000 for OE120 at 200 *m*/*z* (see the Supporting Information for the stage parameters).

**Figure 1:**
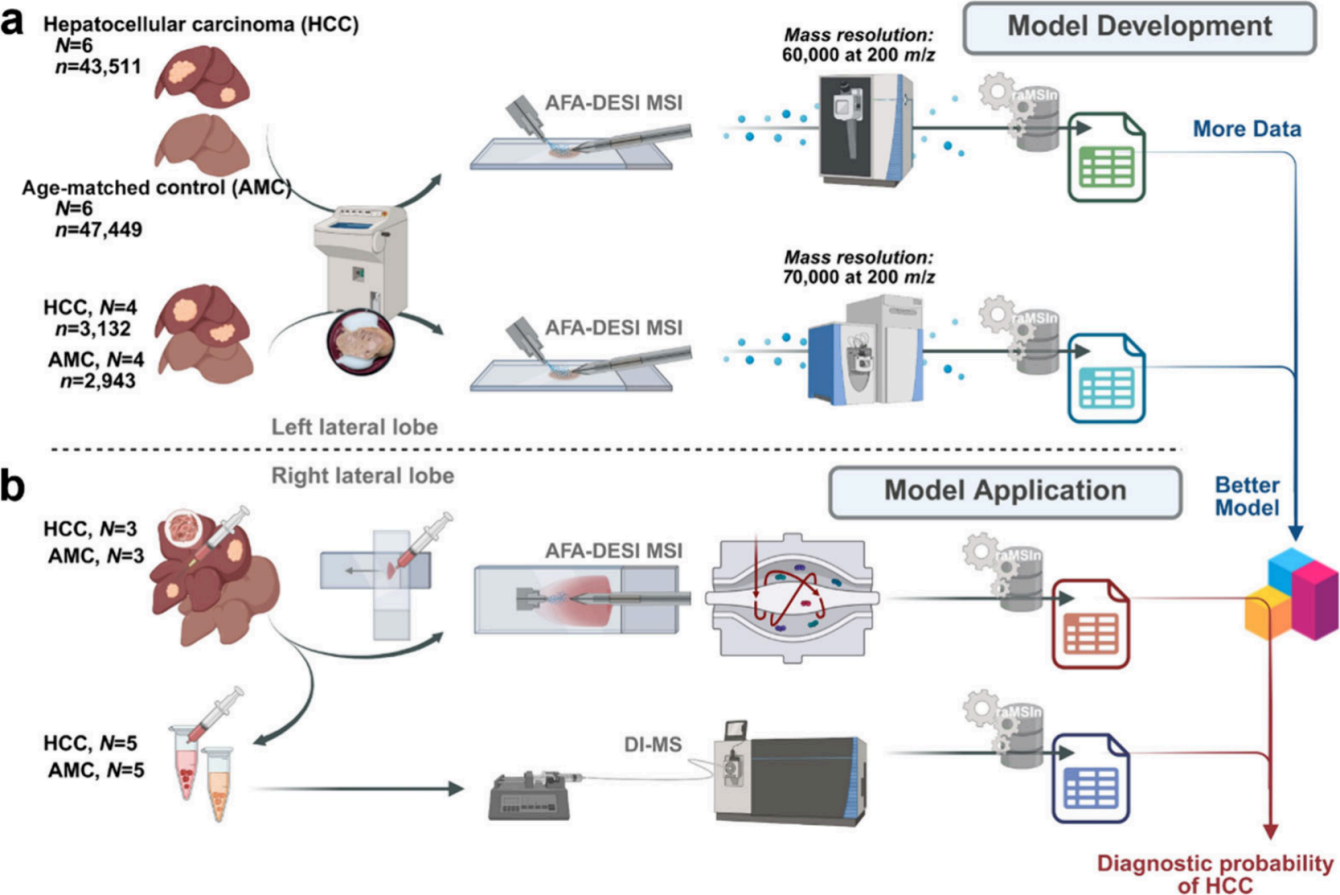
Workflow for multiplatform MS data integration for hepatocellular carcinoma modeling. (a) Model development. A machine learning (ML) model was developed by integrating 2 independent batches of airflow-assisted desorption electrospray ionization MS imaging (AFA-DESI-MSI) data. These data were acquired from different stage parameters and MS platforms, focusing on left liver cryosections from 6 pairs (batch 1) and 4 pairs (batch 2) of hepatocellular carcinoma (HCC) mice and their age-matched controls (AMCs). (b) Model application. Fine-needle aspiration (FNA) biopsies were randomly sampled from the nontumorous regions in the right liver. These samples were used to prepare glass smears (for MSI, 3 HCC/AMC pairs) and liver extracts (for direct infusion, DI-MS, 5 HCC/AMC pairs). The acquired data were utilized to validate the developed model. Feature engineering was performed using resolution-adaptive MS data integration (raMSIn).

### Electrospray Ionization MS

To compare bucket quality by using different data binning approaches, 49 mixed standard solutions at 1000, 500, and 250 ppb were analyzed on a Q-Exactive Focus Orbitrap mass spectrometer (QE Focus, Thermo Fisher Scientific, San Jose, CA, USA). Besides binning quality comparison, for predictive model validation and comparison, a set of DI/ESI-MS data for liver extracts were collected (Figure 1b). Data were acquired in negative ion full-scan mode across the *m*/*z* range of 100–1000 in profile mode at a resolution of 70,000 at 200 *m*/*z* (see the Supporting Information for the optimized ESI source parameters). Five HCC/AMC pairs of diluted liver extracts were introduced using a 500 μL Hamilton syringe at a rate of 50 μL/min. Data acquisition began 2 min after infusion started and continued for 2.7 min, capturing ∼600 scans.

### Modeling and Evaluation Metrics

Data preprocessing procedures, including database construction, denoising, data extraction, transformation, feature selection, and model selection, are detailed in the Supporting Information. An ingested training data set was prepared by integrating 2 independent batches of MSI data (batch 1: 90,960 pixels from 12 cryosections; batch 2: 6075 pixels from 8 cryosections) for model training. Two independent data sets, including glass smear MSI data (88,701 pixels from 6 smears) and DI-MS data (6057 scans from 10 injections), were adopted for model validation (Figure 1).

The predictive performance of logistic regression (LogReg), linear support vector machines (LinearSVC), gradient boosting (GB), eXtreme Gradient Boosting (XGB),^29^ decision tree (DT), and random fortress (RF) modeling was compared primarily using the weighted *F*1 score and secondarily by the Matthews correlation coefficient (MCC) for MSI data, and by recall and precision for DI-MS data (see files 1–6 at 10.5281/zenodo.14962745 for training records). The calculations for the *F*1 score, MCC, recall, and precision are provided in eqs 1–4.

1 $$F1=\frac{TP}{TP+\frac{1}{2}\left(FP+FN\right)}$$

2 $$\mathrm{MCC}=\frac{TP\times TN-FP\times FN}{\sqrt{\left(TP+FP\right)\left(TP+FN\right)\left(TN+FP\right)\left(TN+FN\right)}}$$

3 $$\mathrm{recall}=\frac{TP}{TP+FN}$$

4 $$\mathrm{precision}=\frac{TP}{TP+FP}$$

### Computations and Code Availability

All scripts were developed in Python v.3.11 on Jupyter Notebook (Anaconda3) and Julia v.1.06 using Visual Studio Code (Microsoft). Scripts for database construction are available upon request. Additional scripts for resolution-adaptive regression equation computation, data preprocessing, analytics, and the developed LinearSVC model for HCC detection are accessible at https://github.com/TommyNHL/raMSIn. Updates to the repository will be published with a citation of the final DOI for this work. Details on computational power and the packages used are available in Table S1.

## Results and Discussion

### Resolution-Adaptive MS Data Binning Approach

In untargeted metabolomics, data are typically acquired in centroided mode due to the narrow full width at half-maximum characteristics of HRMS data.^30^ Electrostatic ion trap mass analyzers, such as (Q-)­Orbitrap, are commonly used for separation-based untargeted metabolomics^31–33^ and ambient MSI.^34,35^ We examined the relationship between mass resolution and *m*/*z* values using various in-house and publicly available Orbitrap MS data sets (Figure S01a). Bent decay curves along the *m*/*z* axis were observed, indicating resolutions for different ions varied across low to high mass ranges (100 to 1500 Da). DI-MS data of diverse sample types also exhibited resolution-dependent curves, with higher resolution settings showing greater tangent slopes (Figure 2a), indicating a predictable dependency between mass resolution and *m*/*z* values.

**Figure 2:**
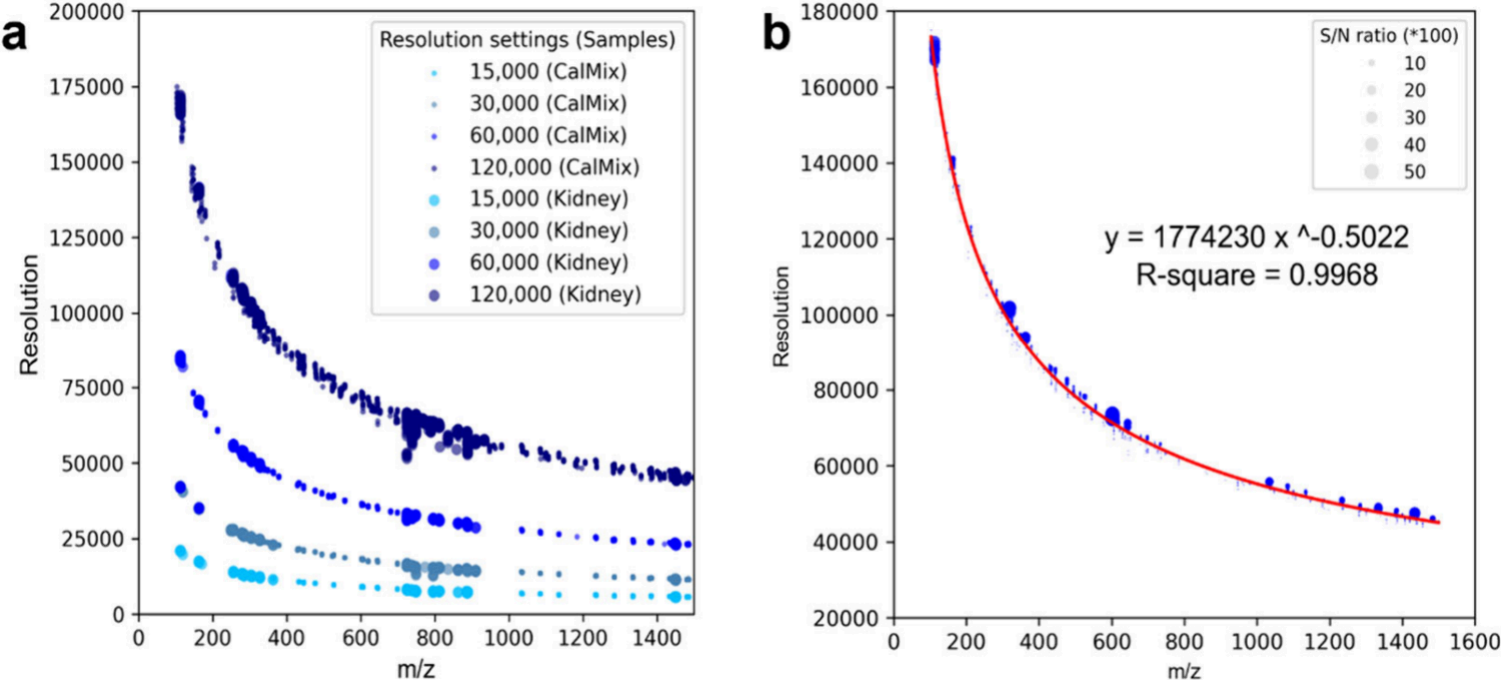
Resolution-adaptive characteristics in Orbitrap MS. (a) A consistent pattern between mass resolution and *m*/*z* values indicated *m*/*z* measurements depend on mass resolution and remain independent of sample type. (b) A regression equation could be computed using a nonlinear least-squares method, exemplifying the data acquired from a direct infusion analysis of a vendor MS calibrant. Abbreviations: CalMix, MS calibrant.

The nonlinear least-squares regression method showed its ability to correlate the dependency, with a coefficient of correlation (*R* ^2^) of 0.9968 that was computed for DI-MS data of the vendor mass spectrometer calibrant (Figure 2b). Similar empirical equations were obtained across various sample introduction methods, including LC-MS (Figure S01b) and multiple row scans from DESI-MSI (Figure S01c). A three-stage protocol was thus developed to calculate resolution-adaptive *m*/*z* bin sizes based on the physical constraint of the Orbitrap.^36^ First, the resolution *R*(Orbi) for a candidate *m*/*z* value was predicted using eq 5, where the coefficient *C*(Orbi) is resolution-aware, and the index *k* is approximately −0.5. Next, the boundaries of the corresponding *m*/*z* bucket were derived from the predicted value of *R*(Orbi). Finally, overlapping buckets were fused to account for mass shifts or the limited resolving power of the mass analyzer. Similar resolution-aware binning strategy should also be applicable to other HRMS mass analyzers, such as FT-ICR (Figure S02) and Q-ToF (Figure S03). However, the discussion on data integration across different mass analyzers is not included in this work due to our limited MS resources.

5 $$R\left(\mathrm{Orbi}\right)\approx C\left(\mathrm{Orbi}\right)\times {\left(m/z\right)}^{k}=\mathrm{centroided apex of mass}/\mathrm{bin size}$$

### MS Data Binning and Integration Performance

MS data binning can cause mass peak aggregation or clipping in a mass bucket instead of representing a single *m*/*z* feature (Figure S04a). Conventional binning approaches, such as constant binning by Dalton values (e.g., 0.1, 0.01, and 0.001 Da),^37–39^ which is adopted in the software mzMine^40^ and MS-DIAL,^41^ and dynamic binning by ppm (e.g., 10 and 5 ppm),^42^ used in the software package XCMS,^43^ were compared. For the low mass region (70–450 *m*/*z*), we found that constant binning, commonly used in MSI data preprocessing, recovered <10% and <54% of ground truth *m*/*z* features by setting 0.1 and 0.01 Da bin sizes, respectively (Figure 3). Dynamic binning by 5 ppm performed comparably well to the resolution-adaptive approach, having a recovery rate of 88–99% for 49 mixed standard solutions at 250, 500, and 1000 ppb. If the boundaries of a *m*/*z* bucket were incorporated with the time domain, mass peak clipping could frequently occur. Hence, extracted ion channels were suggested to be constructed with respect to the whole LC runtime rather than a time window.

**Figure 3:**
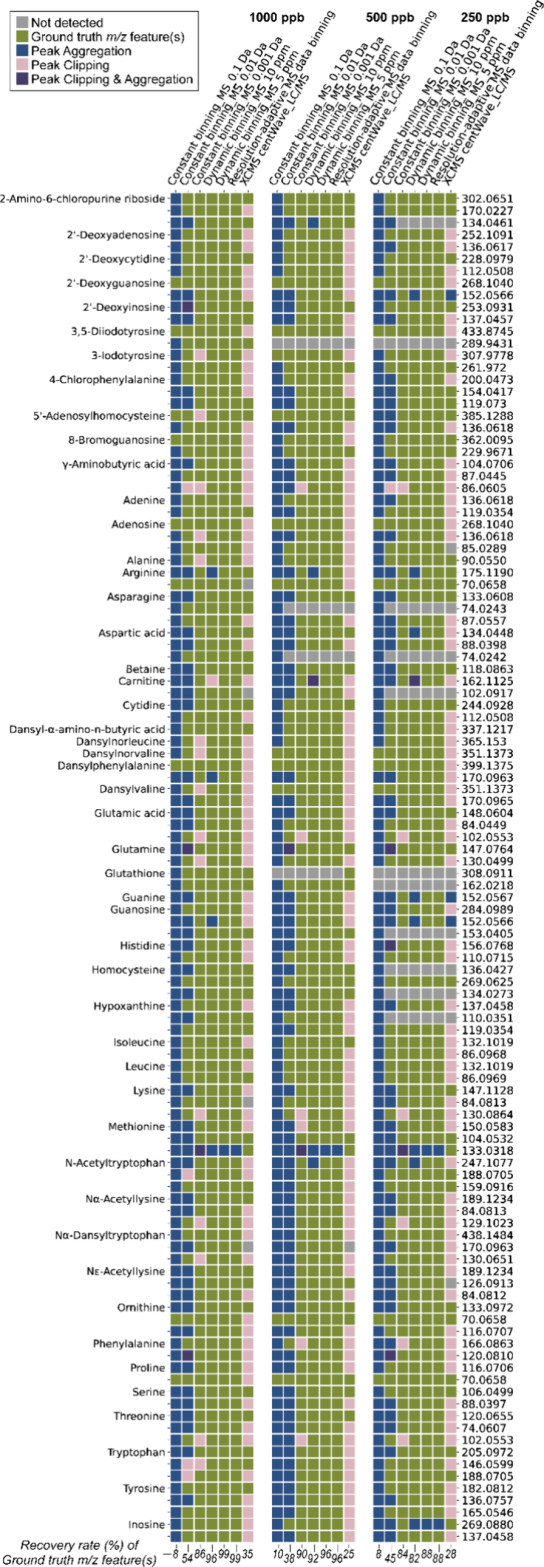
Chemical recovery analysis for 49 metabolites and 112 ground truth *m*/*z* features in 1000, 500, and 250 ppb standard solutions.

Besides the low mass range, dynamic binning performed better than constant binning in the mid (450–900 *m*/*z*) and high (900–1500 *m*/*z*) mass ranges (Figures 4 and S04b,c). Larger bin sizes (0.1 or 0.01 Da) in the low mass range exaggerated differences between upper and lower boundaries, leading to improper mass peak aggregation (Figures 4, S04b,c, and S05). Conversely, narrower bin sizes (0.01 or 0.001 Da) in the high mass range resulted in peak clipping. The performance of dynamic binning demonstrated a broader functional range but remained dependent on the mass range; higher *m*/*z* tolerance could lead to feature aggregation in the low mass range, while lower tolerance could disintegrate *m*/*z* features in the high mass range. In contrast, the resolution-adaptive binning strategy performed the most stably across low, mid, and high mass regions before (Figure S04b,c) and after external data integration (Figure 4).

**Figure 4:**
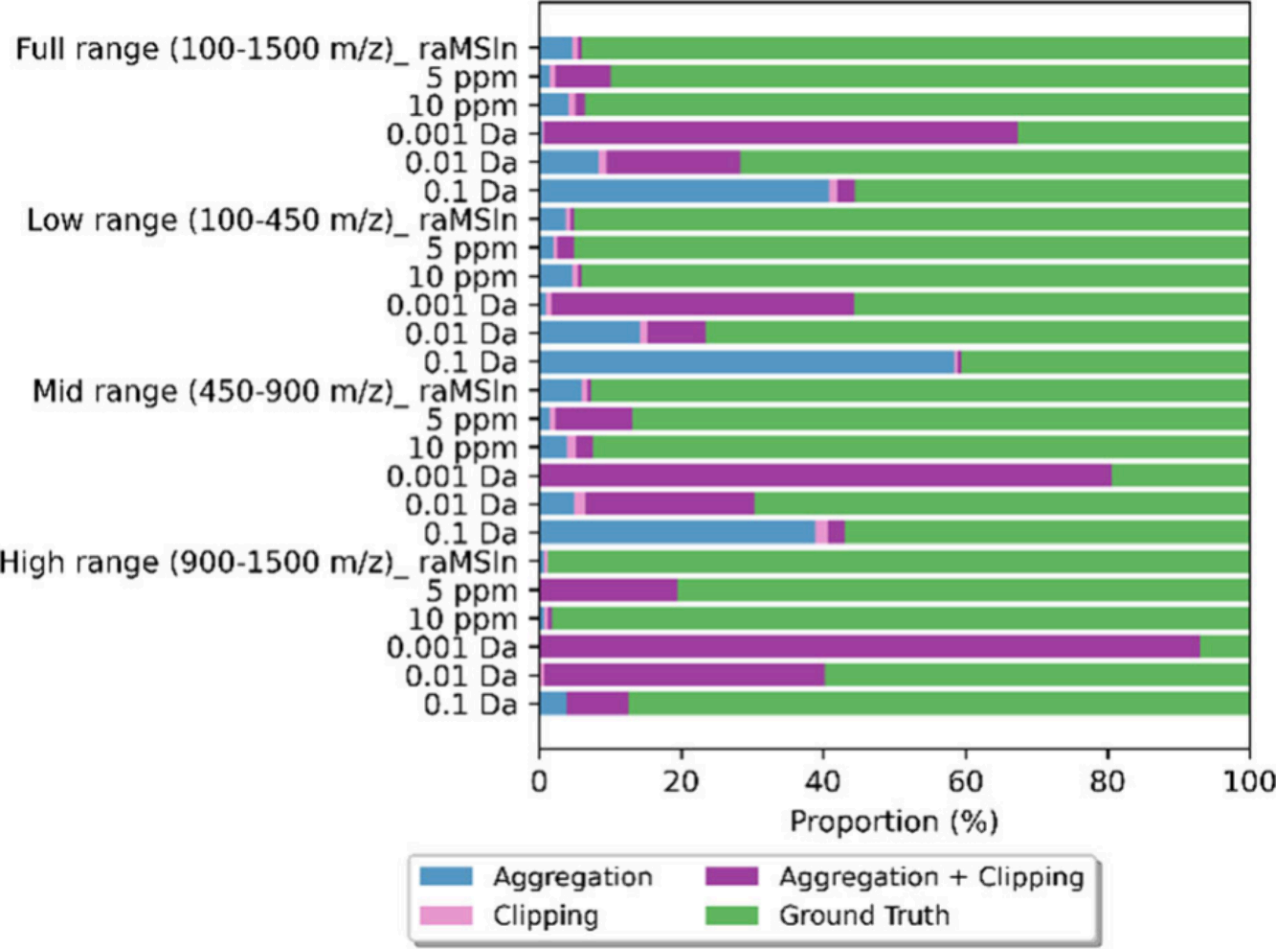
MS data binning performance of different data binning approaches in the low (100–450 *m*/*z*), mid (450–900 *m*/*z*), and high (900–1500 *m*/*z*) mass ranges.

### Resolution-Adaptive Binning Reveals Unique Biological Insight and Better Model Scalability upon Multibatch Data Integration

Data integration increases the size of the training data set, which may enhance predictive model performance. However, using less precise binned data for training and validation can introduce error that propagates along downstream data analytics, introducing uncertainty into predictions.^24^ We compared the ranking of feature importance derived by different binning approaches and found that at least 3 were missed by employing the conventional binning approaches within the 17 significant ground truth *m*/*z* features mined (Table 1). Besides the top 2–3 highest ranked features, the remaining were different in order of importance, implying that the choice of data binning approach can affect feature importance and thus feature selection.

**Table 1:** Comparison of Different Binning Approaches for Feature Importance Ranking

*m*/*z* Features	raMSIn	5 ppm	10 ppm	0.001 Da	0.01 Da	0.1 Da
311.1684^a^	1	1	1	1	1	1
269.2486^a^	2	2	2	2	2	2
215.0328^a^	3	3	3	3	4	>17
295.2278^a^	4	5	10	5	7	>17
883.5331^a^	5	8	16	8	18	>17
309.1704^a^	6	9	6	9	10	>17
738.5059^a^	7	>17	7	>19	6	6
435.2965^a^	8	6	5	6	5	3
280.2361	9	>17	>22	>19	16	>17
241.2173	10	4	8	4	8	5
311.2228	11	>17	>22	>19	>21	>17
339.1996	12	10	13	10	13	13
353.2005	13	>17	>22	>19	3	>17
325.1842	14	17	11	17	14	>17
250.1449^a^	15	>17	9	>19	>21	>17
514.2846^a^	16	>17	21	>19	>21	>17
265.1478	17	13	14	12	17	>17

a Features that are selected for modeling upon redundancy analysis.

To illustrate how model scalability was influenced by selecting different features, models were trained on various batch sizes, and their predictive powers were compared. For the nontrained data used for model validation, such as the smear MSI and DI-MS data, interbatch data integration resulted in a better model by the resolution-adaptive approach and binning by 5 ppm (Table 2). While using dynamic binning MS data integration (dbMSIn) with 10 ppm and constant binning MS data integration (cbMSIn), combining more data did not indicate models with higher predictive power.

**Table 2:** Comparison of Different Binning Approaches for MS Data Integration

Data Sets	Ingested Training^a^		Smear MSI^b^		Direct Infusion^c^			
Metrics	*F*1	MCC	*F*1	MCC	*F*1	MCC	Recall	Precision
Resolution-Adaptive MS Data Integration (raMSIn)								
Model								
LinearSVC (10:10)^d^	0.87	0.73	0.80	0.58	0.74	0.40	0.89	0.63
LinearSVC (6:6)^e^	0.91	0.83	0.76	0.58	0.42	–0.03	0.37	0.48
LinearSVC (4:4)^f^	0.70	0.32	0.62	0.18	0.56	0.03	0.62	0.51
Dynamic Binning MS Data Integration: 5 ppm								
LinearSVC (10:10)^d^	0.91	0.83	0.71	0.37	0.58	0.14	0.60	0.57
LinearSVC (6:6)^e^	0.93	0.85	0.69	0.44	0.45	–0.04	0.42	0.48
LinearSVC (4:4)^f^	0.74	0.43	0.62	0.28	0.39	–0.22	0.39	0.39
Dynamic Binning MS Data Integration: 10 ppm								
LinearSVC (10:10)^d^	0.91	0.83	0.79	0.57	0.56	0.14	0.55	0.57
LinearSVC (6:6)^e^	0.89	0.79	0.62	0.21	0.67	0.30	0.72	0.63
LinearSVC (4:4)^f^	0.74	0.46	0.69	0.30	0.65	0.27	0.69	0.62
Constant Binning MS Data Integration: 0.001 Da								
LinearSVC (10:10)^d^	0.93	0.85	0.73	0.39	0.63	0.21	0.69	0.59
LinearSVC (6:6)^e^	0.94	0.87	0.68	0.33	0.64	0.23	0.68	0.60
LinearSVC (4:4)^f^	0.82	0.62	0.73	0.48	0.71	0.40	0.75	0.68
Constant Binning MS Data Integration: 0.01 Da								
LinearSVC (10:10)^d^	0.91	0.82	0.82	0.64	0.48	–0.09	0.50	0.46
LinearSVC (6:6)^e^	0.92	0.84	0.78	0.59	0.50	0.05	0.48	0.53
LinearSVC (4:4)^f^	0.74	0.48	0.75	0.57	0.60	0.23	0.58	0.62
Constant Binning MS Data Integration: 0.1 Da								
LinearSVC (10:10)^d^	0.85	0.74	0.79	0.58	0.44	–0.09	0.43	0.45
LinearSVC (6:6)^e^	0.88	0.77	0.76	0.49	0.34	–0.32	0.34	0.34
LinearSVC (4:4)^f^	0.81	0.63	0.83	0.67	0.56	0.18	0.53	0.60

a Ingested training data set was prepared by integrating 2 independent batches of desorption electrospray ionization mass spectrometry imaging (DESI-MSI) data of cryosection tissue samples (training batch, *N* = 12, *n* = 90,960; external batch, *N* = 8, *n* = 6075; total: 20 animal subjects and 97,035 MS1 pixels of shotgun analysis).

b A nontrained testing data set that was prepared from an independent batch of DESI-MSI shotgun data of glass smears. Fine-needle aspirate samples were collected at the loci outside tumor regions (*N* = 6, *n* = 88,701).

c Another nontrained testing data set that was prepared from an independent batch of direct infusion MS1 data of tissue extract. Fine-needle aspirate samples were collected at the loci outside tumor regions (*N* = 10, *n* = 6057).

d Model was trained by *n* = 90,960 from *N* = 12 and *n* = 6075 from *N* = 8 [total: 10 animal subjects belonging to the hepatocellular carcinoma (HCC) model and 10 animal subjects belonging to the age-matched controls (AMCs)].

e Model was trained by *n* = 90,960 from *N* = 12 (total: 6 animal subjects belonging to the HCC model and 6 animal subjects belonging to the AMCs).

f Model was trained by *n* = 6075 from *N* = 8 (total: 4 animal subjects belonging to the HCC model and 4 animal subjects belonging to the AMCs).

If given that the selected features were the same, both cbMSIn and dbMSIn resulted in a better model performance (Table S2). Nonetheless, the recall of the model in a smaller scale (i.e., trained from the 4:4 HCC/AMC data) lowered when using cbMSIn with 0.001 Da. This decrease may be attributed to feature clipping occurring for both the ingested training and DI data with *m*/*z* features in the mid and high mass regions (Figure S06). Similarly, when cbMSIn with 0.1 Da was adopted, all 10 discriminative features were aggregated (Figure S06). In the case of dbMSIn at 10 ppm, the *m*/*z* variable 215.0328 was aggregated. In contrast, the resolution-adaptive approach outperformed conventional binning methods, achieving the highest weighted *F*1 score (0.80), recall (0.89), and precision (0.63) for the smear MSI (Figure 5a) and DI-MS (Figure 5b) data, respectively, demonstrating its capability of enhancing model scalability.

**Figure 5:**
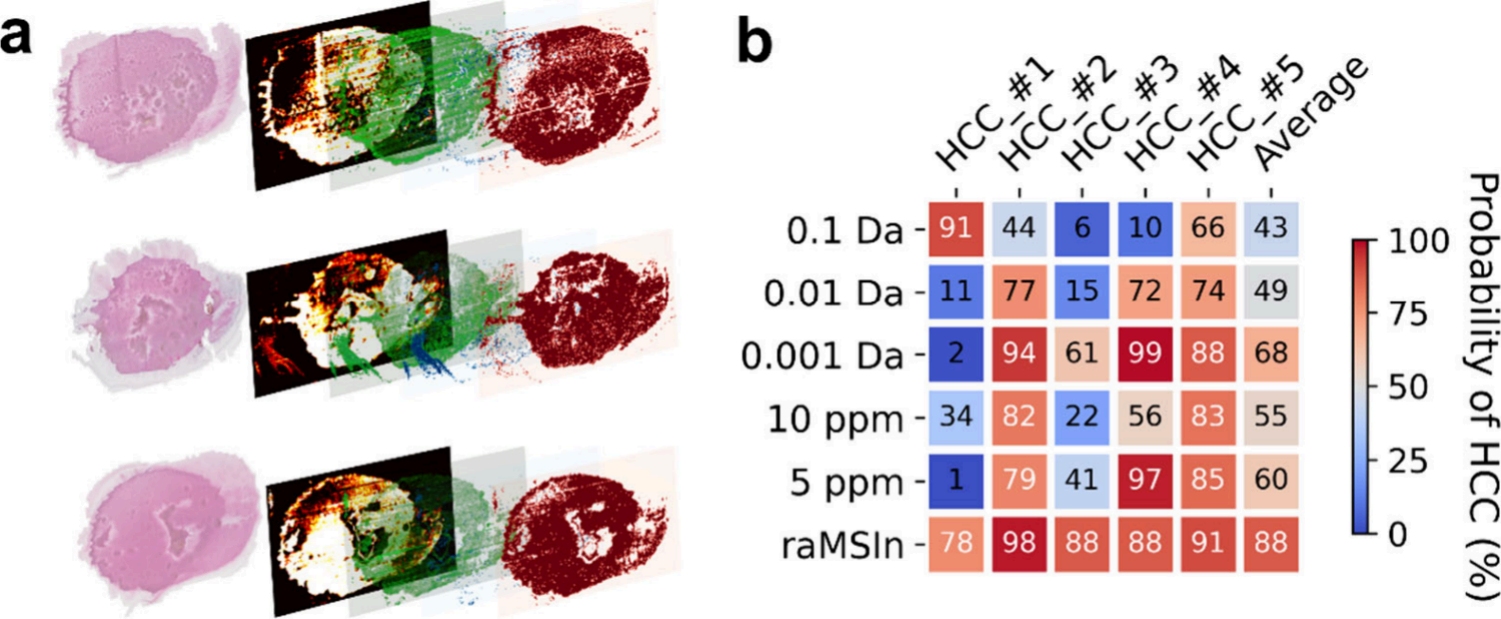
Model application for hepatocellular carcinoma (HCC) determination. (a) Deployment of the model attained by resolution-adaptive MS data binning and integration approach to the smear MS imaging (MSI) data of HCC mouse samples. Data visualization by multiple layers: a post-MSI tissue section stained by hematoxylin and eosin, a region-of-interest filter based on a signal intensity threshold of a *m*/*z* variable 885.5499, a 10-channel ion stacking image, and the spatial distributions of pixels predicted to be normal age-matched controls (AMCs, colored in blue) and those determined as hepatocellular carcinoma (HCC)-diseased (colored in red). (b) Resolution-adaptive binning and its comparison to the conventional data binning approaches for direct infusion analysis of HCC.

### Resolution-Adaptive Binning Reveals Better Model Transferability for Rapid Direct Infusion MS Analysis

Models developed by different data binning approaches were deployed to the nontrained smear MSI and DI-MS data sets for assessing model transferability. The model based on the resolution-adaptive strategy reached the highest disease detection power, showing 88% on average (Figure 5b), which was equivalent to an at least 29% increment compared to the conventional binning approaches. This highlights the potential for the superior versatility of this data binning approach for multiplatform data integration and their generalizabilities for shotgun data analyses by diverse sample introduction methods.

## Conclusions

MS data binning often depends on user-defined settings for bin size, neglecting the nonlinear relationship between mass resolution and *m*/*z* value. A key question remains: is the machine accurately “learning” from complex MS data? Using aggregated or disintegrated *m*/*z* variables to train or validate ML models can compromise the credibility of the predictive models, affecting their scalability and transferability. In this study, we introduce a mass resolution-adaptive data binning approach that leverages physical constricts to bucket *m*/*z* features. This tool effectively addresses mass shifts and integrates MS data from various sample batches and platforms, reducing error propagation to the downstream data analytics and ML modeling.

## Supplementary Material



## Data Availability

All MS raw data used for ML modeling and histograms of *m*/*z* buckets are zipped with the password (raMSIn) and are available online (see 10.5281/zenodo.14962745).

## Notes

Technical details of structural identification, MSI stage parameters, ESI source parameters, data preprocessing methods, the significant *m*/*z* features, and post-MSI tissue staining techniques; figures illustrating the relationship between mass resolution and *m*/*z* values in different sample introduction methods, sample types, and mass analyzers; figures presenting the performance difference among different data binning methods; figures discussing data preprocessing methods; tables listing out the computational resources; comparison of different binning approaches for MS data integration if given the selected features; annotated discriminative metabolites that contributed to HCC prediction; and the sources of the HRMS data (PDF)
