# Supplementary material for: Resolution-Adaptive Binning Enhances Machine Learning Modeling by Interbatch and Multiplatform Orbitrap-Based Shotgun Mass Spectrometry Data Integration
Source: Anal Chem. 2025 Nov 25;97(48):26877–85. doi: 10.1021/acs.analchem.5c05874 (PMC12874219; doi:10.1021/acs.analchem.5c05874)
Supplement: Supplementary file 1 [file ac5c05874_si_001.pdf]

Supporting Information for

Resolution-Adaptive Binning Enhances Machine Learning Modeling By Inter-Batch and Multi-Platform Orbitrap-Based Shotgun Mass Spectrometry Data Integration

Hiu-Lok Ngan,[1,#] Jialing Zhang,[1,#] Kenneth Kin-Leung Kwan,[2,3] Jacinth Wing-Sum Cheu,[2,3] Li Zhong,[1] Yike Guo,[4,5] Xian Yang,[4,6] Carmen Chak-Lui Wong,[2,3,*] Hong Yan,[1,7,*] Zongwei Cai[1,8,*]

[1]State Key Laboratory of Environmental and Biological Analysis, Department of Chemistry, Hong Kong Baptist University, Hong Kong 999077 P. R. China

[2]State Key Laboratory of Liver Research, Department of Pathology, Li Ka Shing Faculty of Medicine, The University of Hong Kong, Hong Kong 999077 P. R. China

[3]Centre for Oncology and Immunology, Hong Kong Science Park, Hong Kong 999077 P. R. China

[4]Department of Computer Science, Hong Kong Baptist University, Hong Kong 999077 P. R. China

[5]Department of Computer Science and Engineering, Hong Kong University of Science and Technology, Hong Kong 999077 P. R. China

[6]Alliance Manchester Business School, The University of Manchester, Manchester M15 6PB United Kingdom

[7]Department of Biology, Hong Kong Baptist University, Hong Kong 999077 P. R. China

[8]Eastern Institute of Technology, Ningbo 315200 P. R. China

[#]The authors contributed equally to this work.

***Corresponding authors: Drs. Carmen Chak-Lui Wong, Hong Yan, and Zongwei Cai**

Emails: carmencl@pathology.hku.hk; hongyan@hkbu.edu.hk; zwcai@hkbu.edu.hk

TABLE OF CONTENTS

ADDITIONAL TEXT S-2

Confidence Levels for Structural Identification S-2

MS Imaging Stage Parameters S-2

ESI Source Parameters S-2

Data Pre-Processing Procedures S-3

Ten Generic Biomarkers Mined by raMSIn from Multiple Orbitrap-Based Platforms for Hepatocellular Carcinoma Detection S-6

Hematoxylin and Eosin (H&E) Staining S-6

References for Supporting Text S-7

ADDITIONAL FIGURES S01–21 .... S-8

Figure S01–S03: The relationship between mass resolution and *m/z* values. S-8

Figure S04–S06: Performance difference among different data binning methods. S-11

Figure S07–S21 : Data pre-processing methods. S-15

ADDITIONAL TABLES S1–S4 .... S-30

Table S1: Computational Power and Packages Used. S-30

Table S2: Summary of the Selected Machine Learning Algorithms' Performance and the Comparison of Different Binning Approaches for MS Data Integration. S-31

Table S3: Summary of 10 Annotated Discriminative Metabolites that Contributed to Hepatocellular Carcinoma Prediction. S-33

Table S4: Sources of the High-Resolution MS Data. S-34

ADDITIONAL TEXT

Confidence Levels for Structural Identification

The confidence level of metabolite annotation follows the Metabolomics Standards Initiative.[1] For level 1 identification confidence, the software *TidyMass* was employed to match the *m/z* values of molecular ion (MS1) and fragments, as well as retention times (RTs) with referenced values recorded in an in-house database.[2] Level 2B annotations of lipids were achieved using LipidSearch (Thermo Fisher Scientific, San Jose, CA, USA). For level 3 confidence, a bulk search of MS1 was performed using the Human Metabolome Database (HMDB)[3] and LIPID MAPS.[4] Candidates with the least absolute mass error were selected and reported.

MS Imaging Stage Parameters

For the Hybrid Q-Exactive Orbitrap mass spectrometer (QE, Thermo Fisher Scientific, San Jose, CA, USA) platform, airflow-assisted desorption electrospray ionization (DESI) imaging platform (AFA-DESI, Beijing Victor, Beijing, China) stage parameters included a vacuum pressure of −50 kPa, spray voltage of −4 kV, and gas pressure of 180 psi. For the Orbitrap Exploris 120 mass spectrometer (OE120, Thermo Fisher Scientific, San Jose, CA, USA) platform, vacuum pressure was set at −60 kPa, with a gas pressure of 140 psi. A histologically compatible solvent system (1:1 ACN/DMF) was employed at flow rates of 3 μL/min for QE and 2 μL/min for OE120.

ESI Source Parameters

The optimized ESI source parameters included a S-lens RF level at 100 units; spray voltage at 3.5 kV (static); sheath gas at 40 units; auxiliary gas at 10 units; sweep gas at 3 units; ion transfer tube temperature at 320 °C; and vaporizer temperature at 350 °C.

Data Pre-Processing Procedures

Prior to feature selection and machine learning (ML) model development, the MSI data were pre-processing using 5 modules: (1) resolution-adaptive regression equation computation, (2) Structured Query Language (SQL) database construction, (3) denoising, data (4) extraction, (5) transformation (the ETL process), (6) feature selection, and (7) model selection. All scripts in this study were written in Python v.3.11 on Jupyter Notebook (Anaconda3) and Julia v.1.06 using Visual Studio Code (Microsoft). The scripts for SQL database construction are available upon request at https://github.com/jialingzhangchem/raMSI. Additionally, scripts for resolution-adaptive regression equation computation, denoising, and data ETL can be found at https://github.com/TommyNHL/raMSIn.

Resolution-Adaptive Regression Equation Computation. To begin, a resolution-adaptive regression equation can be computed using a developed graphical user interface, as shown in **Figure S07**. This can be accomplished with the publicly available Jupyter Notebook named "NOV17_GUI_trial.ipynb" found in the directory "A_GUI4regression". If one performs MS calibration with a vendor MS calibrant before MSI, the acquired direct infusion data can be used to compute the regression equation by selecting "Import a .raw data" (**Figure S07a**). To address mass shifts during the MSI of a batch of samples, multiple .raw files can be pre-processed by selecting "Import a batch of .raw data" (**Figures S07a** and **S07b**). After file selection, clicking "Run" to wait until a scatter plot is generated (**Figure S07c**). The parameters of the regression equation can be copied to the clipboard by clicking the button named "Copy the regression equation into Clipboard" (**Figure S07c**). The parameters are returned as the datatype String as shown in **Figure S07d**.

Structured Query Language (SQL) Database Construction. Database construction can be achieved by pasting the returned parameters of the calculated regression equation into a private Jupyter Notebook named "raMSI.ipynb". The implementation of this notebook requires

the installation of Apache Spark^{TM}. Using “raMSI.ipynb”, the imported MS data can be organized into tabular format. Firstly, a list of *m/z* variables with calculated upper and lower *m/z* boundaries is generated. Secondly, if there are overlapping *m/z* buckets, mass infusion is employed to merge these *m/z* buckets. Thirdly, the variables are filtered to include only those with a signal-to-noise ratio greater than 10 in at least 99% of pixels across a batch of MSI data with pre-annotated label “1” (for hepatocellular carcinoma samples, HCC) and “0” (for age-matched controls, AMC). Finally, the resultant *m/z* variables are arranged as columns, with pixel sample queries organized as rows.

Denoising. Denoising is achieved through dimensionality reduction of the constructed dataframe using the publicly available Jupyter Notebook named “C_Denoise_2_2_UMAP.ipynb”. We employed Uniform Manifold Approximation and Projection (UMAP) combined with precomputed k-nearest neighbors for dimensionality reduction, evaluating the k-value using the silhouette score (**Figure S08**). A clustering into 4 groups was suggested based on the highest silhouette score (>0.45). Hyperparameter selection was then performed to optimize clustering by UMAP (**Figure S09**). A minimum distance of 0.05 and number of neighbors of 5 were finally selected. We previewed the ion image belonging to the green cluster in the optimal UMAP projection. It is revealed that the ion images belonging to the green cluster represents signal ions, showing higher signal intensity in tissue regions compared to background areas. Therefore, only a cluster of *m/z* features represented by the green dots were filtered in for data ETL.

Data Extract, Transform, and Load. Data of the *m/z* variables with the highest importance are extracted for transformation. Using the publicly available Jupyter Notebook named “D_ML_Model_Importance_2_2.ipynb”, hyperparameter selection was performed to optimize an eXtreme Gradient Boosting (XGB) model.[5] Feature selection was assessed using the average

area under the receiver operating characteristics (AUROC) curve through 5-fold cross-validation of the XGB model, based on resolution-aware features. The *m/z* features were then ranked based on their importance scores (**Figure S10a**). Our sensitivity analysis revealed that a model constructed from 17 features demonstrated considerable robustness (**Figure S10b**). The extracted data were transformed using the publicly available script written in Julia, namely "E1_PreEDA.jl". The balance data were scaled and transformed by square root and normalized by z-score. The resultant dataset is now ready to load for feature selection and ML model optimization and selection.

Feature Selection. Feature redundancy was then assessed among these 17 candidates across various datasets, including training, external, FNA glass smear MSI, and direct infusion datasets (**Figures S11a–S11d**). Pearson correlation was utilized to remove redundant features using the publicly available Jupyter Notebook named "F1_FeatureSelection.ipynb". Those with higher importance in each ion pair when the coefficient of Pearson correlation *r* value exceeded 0.80 were retained. Ten features were retained as the generic HCC diagnostic biomarkers. The details of these 10 annotated discriminative features are provided in **Table S3**. Same procedures were performed for the conventional data binning methods for feature selection (**Figures S12–S21**).

Model Selection. Before comparing the performance of models developed using different binning methods, model selection was conducted. Predictions made using LogReg, LinearSVC, and GB models on smear MSI dataset demonstrated greater accuracy ($F1 \geq 0.75$). In contrast, XGB, DT, and RF models performed less effectively. Recognizing that different algorithms may exhibit selective robustness to specific datasets, a LinearSVG was selected as it presents greatest robustness and transferability across various datasets. The model is attained by L2 regularization with a regularization parameter of C (402) using hinge loss function.

Ten Generic Biomarkers Mined by raMSIn from Multiple Orbitrap-Based Platforms for Hepatocellular Carcinoma Detection

To evaluate the scalability of models developed using different MS data binning approaches, 10 significant biomarkers for multi-platform HCC diagnosis using raMSIn in advance (**Table S3**). Among the mined features, the most significant one was either 13(S)- or 9(S)- HpODE, both derivatives of linoleic acid and endogenous lipid hydroperoxides. Their decomposition is linked to the generation of reactive lipid alkyl radicals and the genotoxic compound 4-hydroxy-nonenal (4-HNE).[6] On the other hand, altered redox status in cancer cells has been also associated with lipid peroxidation caused by 4-HNE.[7] In our HCC mouse model, we observed that fatty acids (FAs) such as FA 17:0, FA 18:1;O, and FA 17:4;O3 were down-regulated, while undecanedioic acid (FA 11:1;O2) was up-regulated. The fatty acid degradation pathway has previously been utilized to develop a molecular classifier for HCC.[8] Furthermore, we found that diacyl lipids, including phosphatidylinositol (PI), phosphatidylethanolamine (PE), and phosphatidylserine (PS), were also up-regulated. Notably, the hepatic bile acid, whose synthesis is linked to fibrosis and HCC progression,[9] was found to be up-regulated.

Hematoxylin and Eosin (H&E) Staining

Due to the use of histological compatible solvents (1:1 acetonitrile/dimethylformamide), the tissue sections after AFA-DESI MSI experiments could still be stained. The H&E staining protocol followed previously established methods.[10] Briefly, the samples were rehydrated in methanol for 2 min and rinsed in tap water for 10 dips. This was followed by 1.5 min in hematoxylin, 10 dips in water, a quick dip in 0.1% ammonia, another 10 dips in water, and then 8 s in eosin. All sections were dehydrated in absolute ethanol and cleared in xylene (6 dips). The stained tissues were visualized using ZEISS Axioscan 7 slide scanner (ZEISS, Oberkochen, Baden-Württemberg, Germany) at 20× and 40× magnifications separately.

References for Supporting Text

(1) Sumner, L. W.; Amberg, A.; Barrett, D.; Beale, M. H.; Beger, R.; Daykin, C. A.; Fan, T. W. M.; Fiehn, O.; Goodacre, R.; Griffin, J. L.; Hankemeier, T.; Hardy, N.; Harnly, J.; Higashi, R.; Kopka, J.; Lane, A. N.; Lindon, J. C.; Marriott, P.; Nicholls, A. W.; Reily, M. D.; Thaden, J. J.; Viant, M. R. Proposed Minimum Reporting Standards for Chemical Analysis: Chemical Analysis Working Group (CAWG) Metabolomics Standards Initiative (MSI). *Metabolomics* **2007**, *3* (3), 211–221.

(2) Shen, X.; Yan, H.; Wang, C.; Gao, P.; Johnson, C. H.; Snyder, M. P. TidyMass an Object-Oriented Reproducible Analysis Framework for LC–MS Data. *Nat Commun* **2022**, *13*, No. 4365.

(3) Wishart, D. S.; Guo, A. C.; Oler, E.; Wang, F.; Anjum, A.; Peters, H.; Dizon, R.; Sayeeda, Z.; Tian, S.; Lee, B. L.; Berjanskii, M.; Mah, R.; Yamamoto, M.; Jovel, J.; Torres-Calzada, C.; Hiebert-Giesbrecht, M.; Lui, V. W.; Varshavi, D.; Varshavi, D.; Allen, D.; Arndt, D.; Khetarpal, N.; Sivakumaran, A.; Harford, K.; Sanford, S.; Yee, K.; Cao, X.; Budinski, Z.; Liigand, J.; Zhang, L.; Zheng, J.; Mandal, R.; Karu, N.; Dambrova, M.; Schiöth, H. B.; Greiner, R.; Gautam, V. HMDB 5.0: The Human Metabolome Database for 2022. *Nucleic Acids Res* **2022**, *50* (D1), D622–D631.

(4) Conroy, M. J.; Andrews, R. M.; Andrews, S.; Cockayne, L.; Dennis, E. A.; Fahy, E.; Gaud, C.; Griffiths, W. J.; Jukes, G.; Kolchin, M.; Mendivelso, K.; Lopez-Clavijo, A. F.; Ready, C.; Subramaniam, S.; O'Donnell, V. B. LIPID MAPS: Update to Databases and Tools for the Lipidomics Community. *Nucleic Acids Res* **2024**, *52* (D1), D1677–D1682.

(5) Chen, T.; Guestrin, C. XGBoost: A Scalable Tree Boosting System. In *Proceedings of the ACM SIGKDD International Conference on Knowledge Discovery and Data Mining*; 2016; Vol. 13-17-August-2016.

(6) Qin, H.; Huang, C. H.; Mao, L.; Xia, H. Y.; Kalyanaraman, B.; Shao, J.; Shan, G. Q.; Zhu, B. Z. Molecular Mechanism of Metal-Independent Decomposition of Lipid Hydroperoxide 13-HPODE by Halogenated Quinoid Carcinogens. *Free Radic Biol Med* **2013**, *63*, 459–466.

(7) Yin, H. The Role of Lipid Peroxidation During the Progression of Human Hepatocellular Carcinoma (HCC). *Free Radic Biol Med* **2018**, *124*, No. 563–564.

(8) Li, B.; Li, Y.; Zhou, H.; Xu, Y.; Cao, Y.; Cheng, C.; Peng, J.; Li, H.; Zhang, L.; Su, K.; Xu, Z.; Hu, Y.; Lu, J.; Lu, Y.; Qian, L.; Wang, Y.; Zhang, Y.; Liu, Q.; Xie, Y.; Guo, S.; Mehal, W. Z.; Yu, D. Multiomics Identifies Metabolic Subtypes Based on Fatty Acid Degradation Allocating Personalized Treatment in Hepatocellular Carcinoma. *Hepatology* **2024**, *79* (2), 289–306.

(9) Gadaleta, R. M.; Scialpi, N.; Peres, C.; Cariello, M.; Ko, B.; Luo, J.; Porru, E.; Roda, A.; Sabbà, C.; Moschetta, A. Suppression of Hepatic Bile Acid Synthesis by a Non-Tumorigenic FGF19 Analogue Protects Mice from Fibrosis and Hepatocarcinogenesis. *Sci Rep* **2018**, *8* (1), No. 17210.

(10) Guo, W.; Shi, Z.; Zeng, T.; He, Y.; Cai, Z.; Zhang, J. Metabolic Study of Aristolochic Acid I-Exposed Mice Liver by Atmospheric Pressure Matrix-Assisted Laser Desorption/Ionization Mass Spectrometry Imaging and Machine Learning. *Talanta* **2022**, *241*, No. 123261.

ADDITIONAL FIGURES

Figure S01

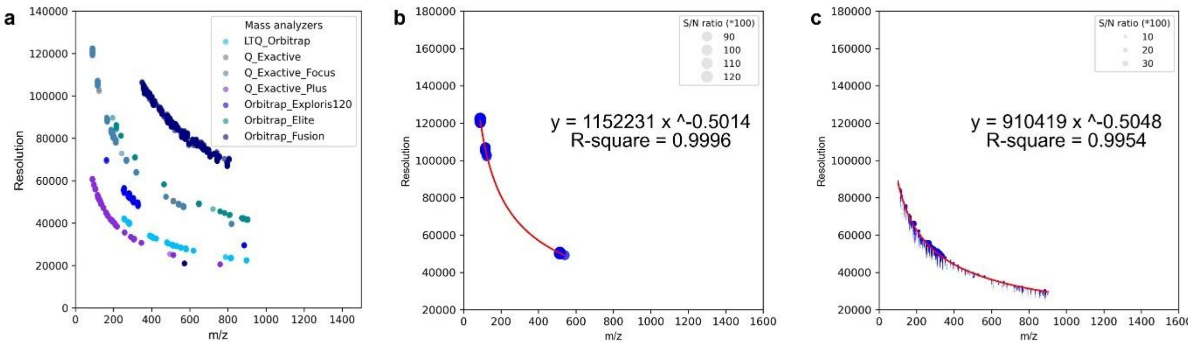

Figure S01: Relationship between mass resolution and *m/z* values. (a) A variety of mass spectrometry (MS) data collected from (Q-)Orbitrap mass analyzers demonstrated a consistent pattern between mass resolution and mass-to-charge ratio (*m/z*) of ions from 100 to 1500 Da. **(b)** Using liquid chromatography-based MS data and **(c)** a multi-row desorption electrospray ionization MS imaging (DESI-MSI) dataset of a whole mouse liver tissue section, the corresponding regression curves could be established to link the data points in a *m/z*-to-resolution plot. The sources of the (Q-)Orbitrap data are detailed in **Table S4**.

Figure S02

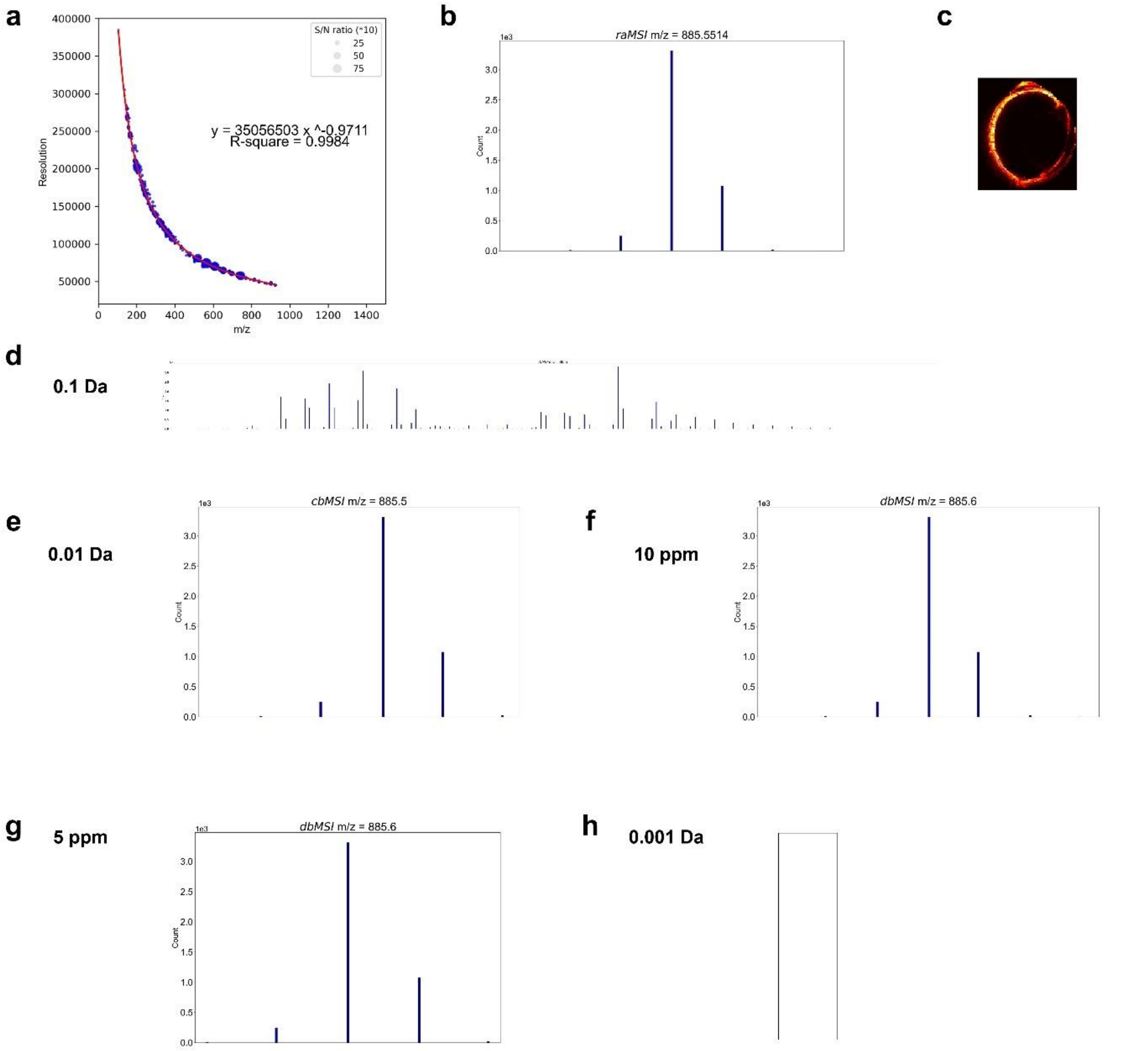

Figure S02: Conventional and resolution-adaptive binning for FT-ICR data. (a) Relationship between mass resolution and *m/z* value in FT-ICR-MS. Data source is detailed in **Table S4**. **(b)** Ion bin of 885.5514 *m/z* defined by resolution-adaptive binning **(c)** and its ion image. **(d)** Feature aggregation occurs by constant binning by 0.1 Da. Bin sizes defined by **(e)** 0.01 Da, **(f)** 10 ppm, and **(g)** 5 ppm are ideal in this case. **(h)** Feature binned by 0.001 Da is absent, most probably because feature clipping occurs, and the derivative bin is low in abundance.

Figure S03

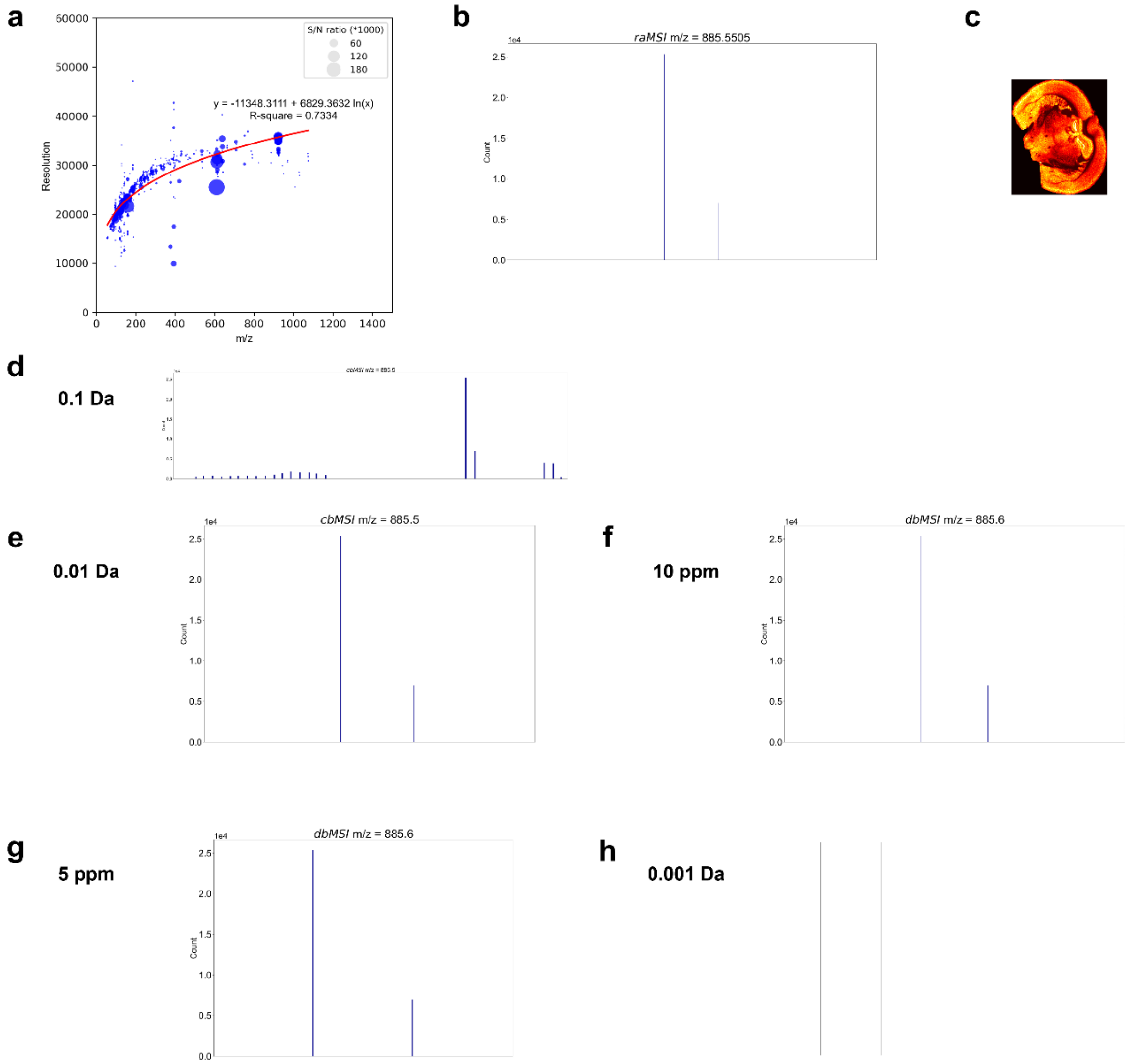

Figure S03: Conventional and resolution-adaptive binning for Q-ToF data. (a) Relationship between mass resolution and *m/z* value in Q-ToF-MS. Data source is detailed in **Table S4**. **(b)** Ion bin of 885.5505 *m/z* defined by resolution-adaptive binning **(c)** and its ion image. **(d)** Feature aggregation occurs by constant binning by 0.1 Da. Bin sizes defined by **(e)** 0.01 Da, **(f)** 10 ppm, and **(g)** 5 ppm are ideal in this case. **(h)** Feature binned by 0.001 Da is absent, most probably because feature clipping occurs, and the derivative bin is low in abundance.

Figure S04

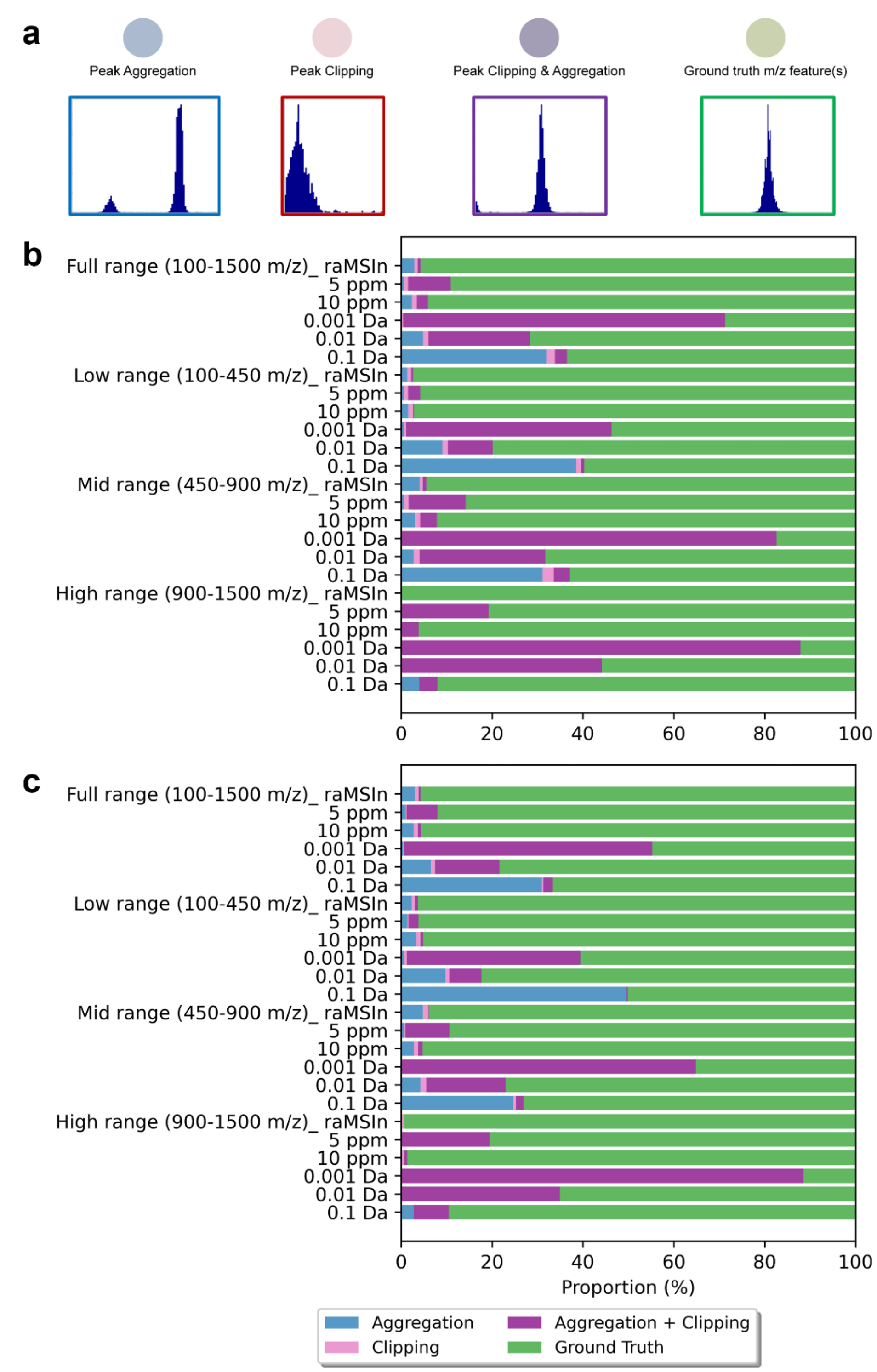

Figure S04: Resolution-adaptive binning and its comparison to the conventional data binning approaches in MS data integration. (a) Coloring scheme for visualizing aggregated, disintegrated, and ideal *m/z* features. Statistics of the binning performance

for the MS imaging (MSI) data from **(b)** batch 1 (6 pairs) and **(c)** batch 2 (4 pairs) hepatocellular carcinoma mice and their age-matched controls.

Figure S05

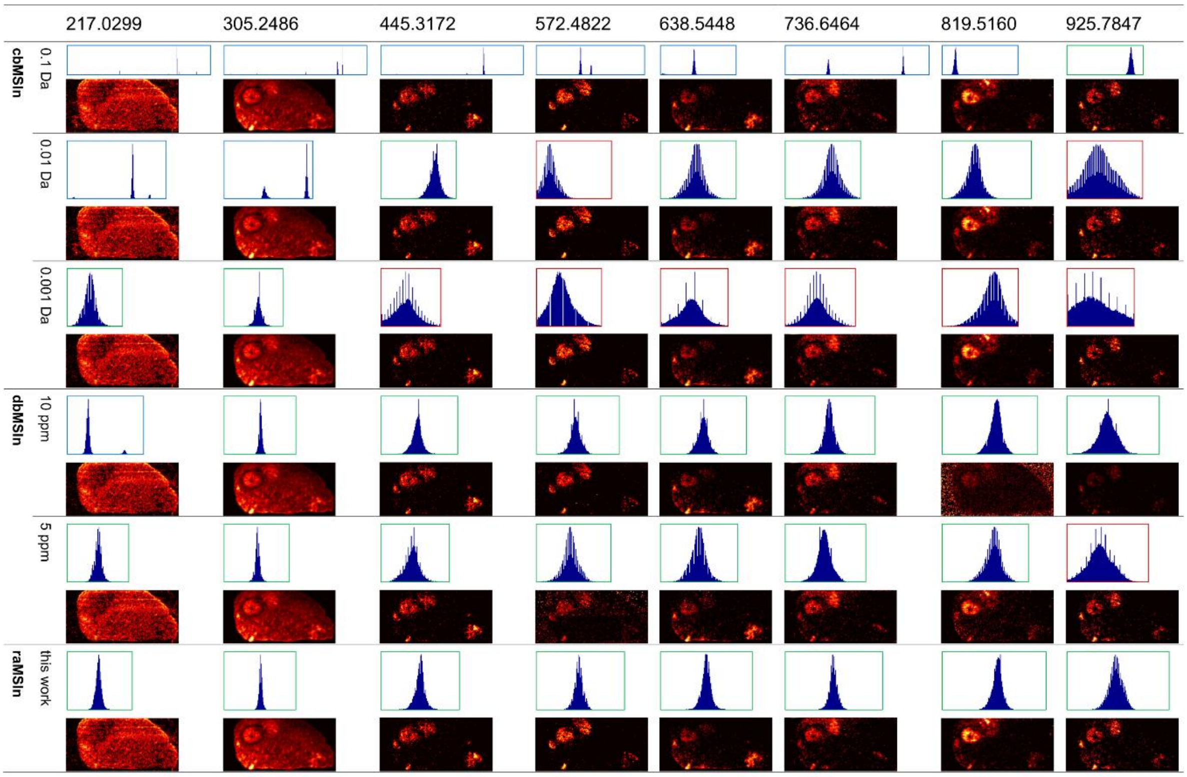

Figure S05: Qualities of the selected features' *m/z* bucket at each 100 *m/z* step and their ion images using different data binning approaches. Features exhibiting critical defects from conventional binning methods were consistently observed along the *m/z* axis for every 100 units. Ideal *m/z* variables are green-colored; Aggregated features are blue-colored; Disintegrated features are red-colored; Features with both issues are purple-colored. *Abbreviations: cbMSIn, Constant binning MS data integration; dbMSIn, Dynamic binning MS data integration; raMSIn, Resolution-adaptive MS data integration.*

Figure S06

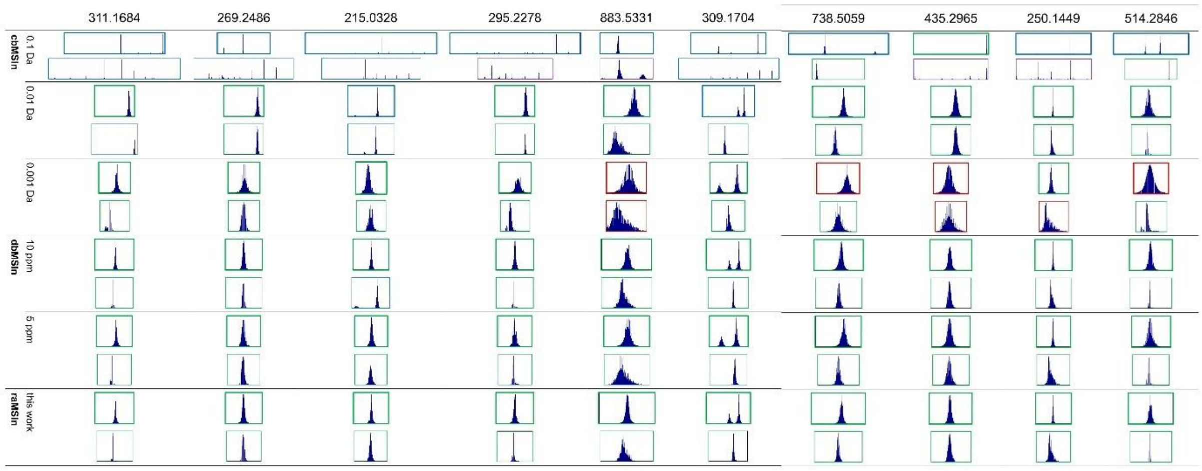

Figure S06: Discriminative features' *m/z* bucket qualities upon data integration using different data binning approaches. Some of the discriminative features selected for model training were found to be aggregated and/or clipped when conventional data integration methods were employed. The *m/z* buckets of the 10 most significant variables produced by resolution-adaptive MS data integration (raMSIn) were optimal for both ingested data (indicated by the upper row with bold grid) and non-ingested flow injection analysis-MS data (indicated by the lower row with narrow grid). The green-colored grid indicated the *m/z* bucket contained only 1 feature without including others; the blue-colored grid represented multiple features that were combined, indicating feature aggregation; the red-colored grid showed features were truncated or lost due to feature clipping; the purple-colored grid annotated both issues (feature aggregation and feature clipping) occurring simultaneously. *Abbreviations: cbMSIn, Constant binning MS data integration; dbMSIn, Dynamic binning MS data integration.*

Figure S07

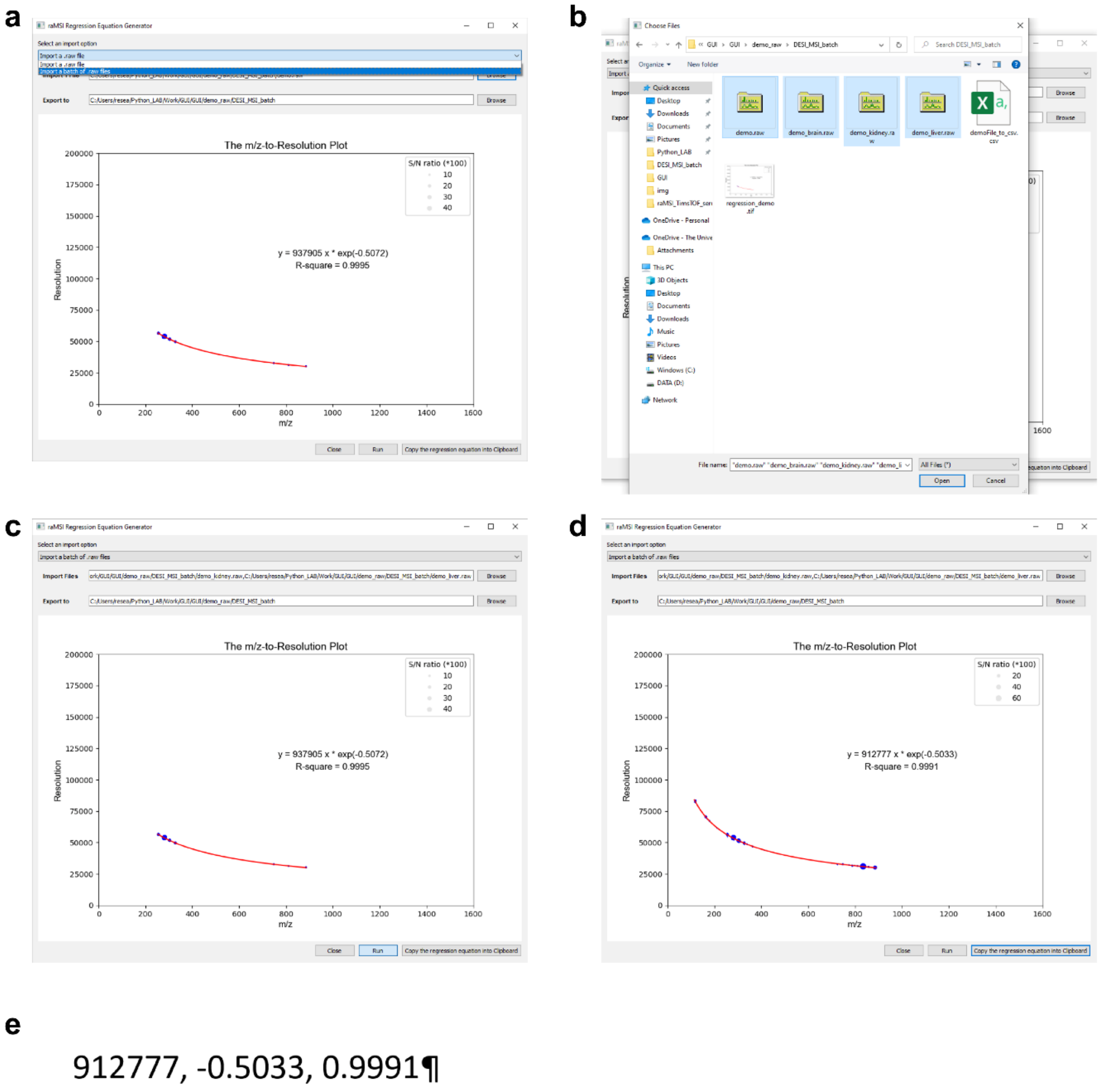

Figure S07: Graphical user interface for regression parameter calculation. (a) Either a single .raw data or a batch of .raw files can be selected for regression. **(b)** After selecting all files used for regression equation determination, click “Open”. **(c)** After browsing or inputting the directory used to save the resulting files, click “Run”. **(d)** A preview of the regression equation and a plot are generated. By clicking the button named “Copy the regression equation into Clipboard”, **(e)** the calculated regression parameters are returned.

Figure S08

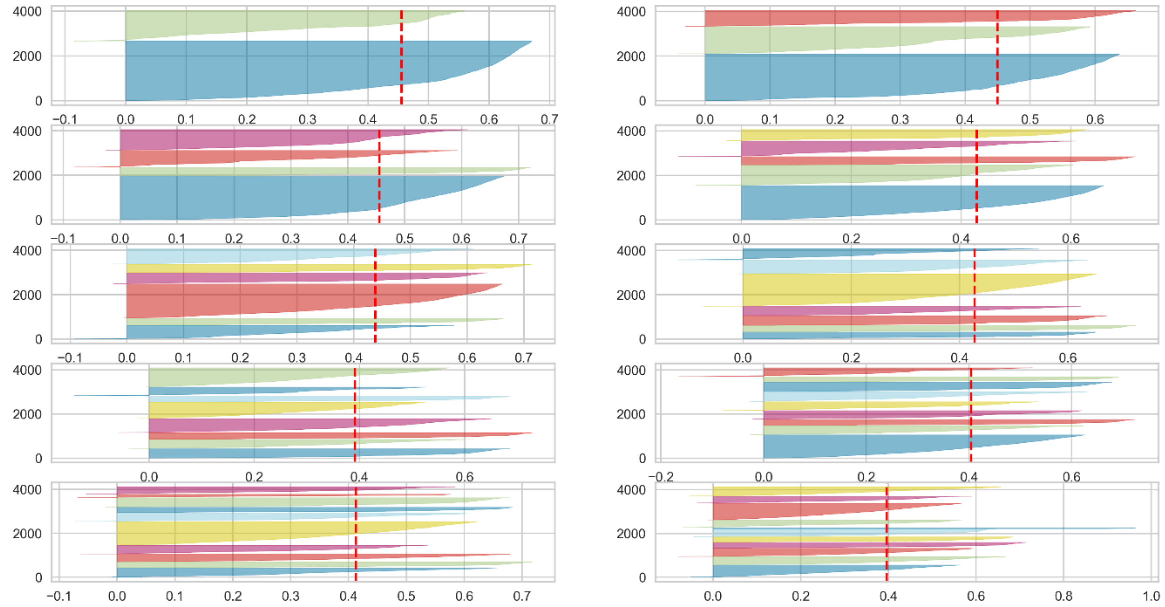

Figure S08: The Silhouette Score. A precomputed k-nearest neighbors for dimensionality reduction by Uniform Manifold Approximation and Projection is computed as 4 due to its highest score (>0.45).

Figure S09

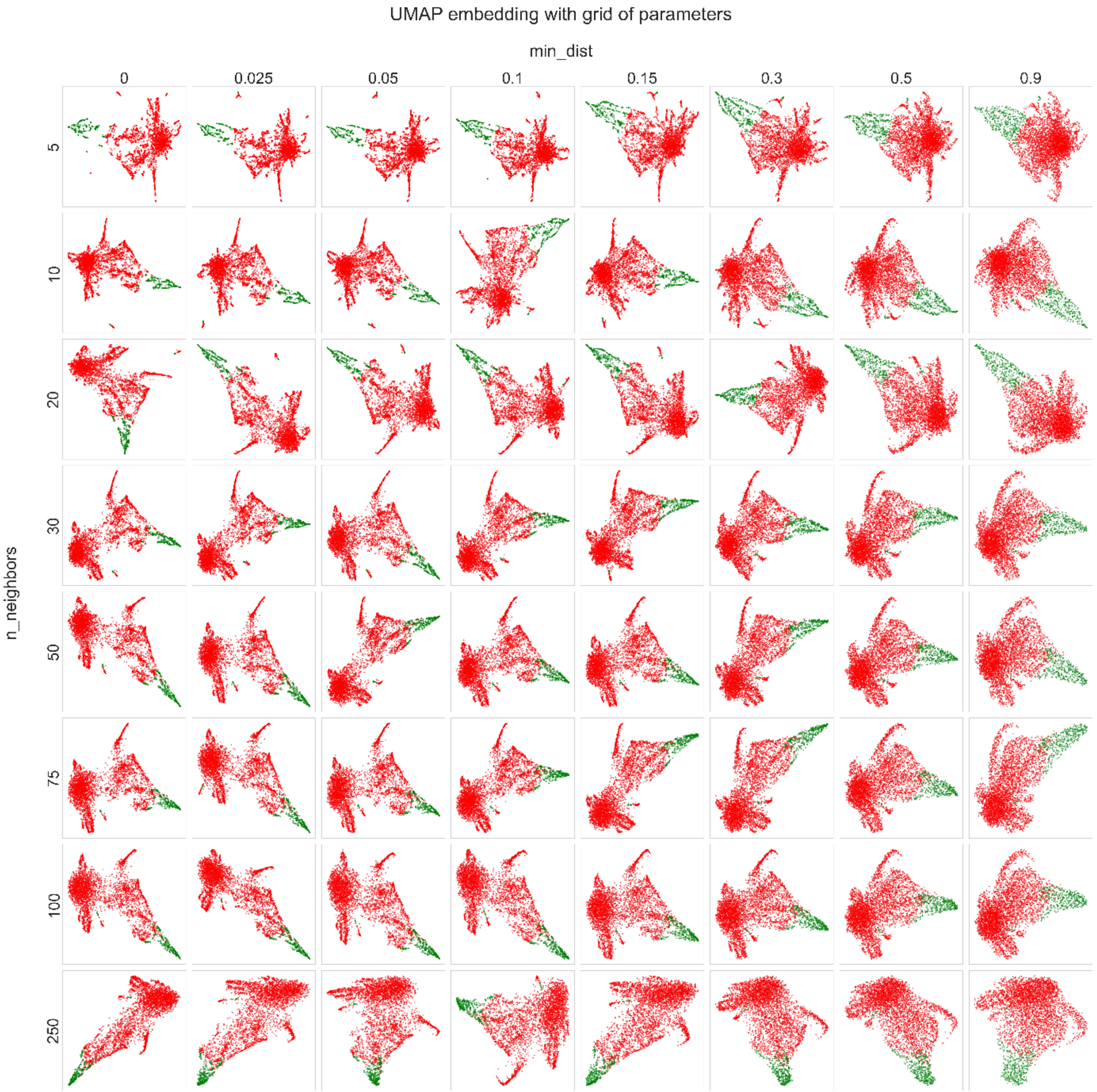

Figure S09: Hyperparameter selection of Uniform Manifold Approximation and Projection. A minimum distance of 0.05 and number of neighbors of 5 were selected.

Figure S10

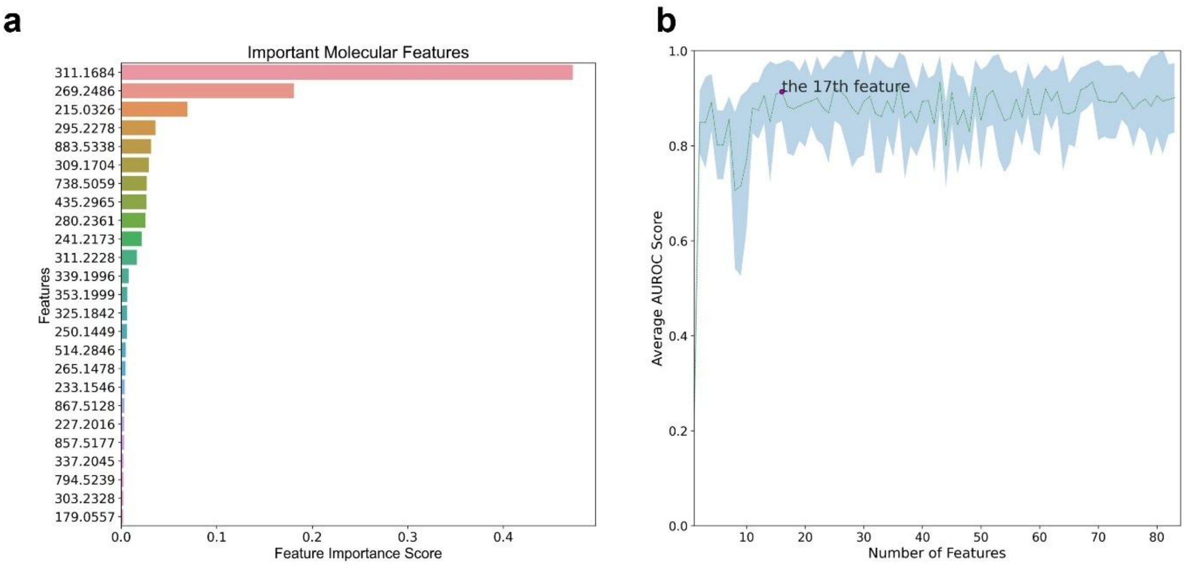

Figure S10: Discriminative feature selection for the resolution-adaptive binning approach. **(a)** Features were ranked based on their feature importance scores derived from the eXtreme Gradient Boosting model. **(b)** Feature selection was performed through model sensitivity analysis. A feature was included if its addition did not lead to 2 consecutive decreases in the average area under the receiver operating characteristics (AUROC) during 5-fold cross-validation.

Figure S11

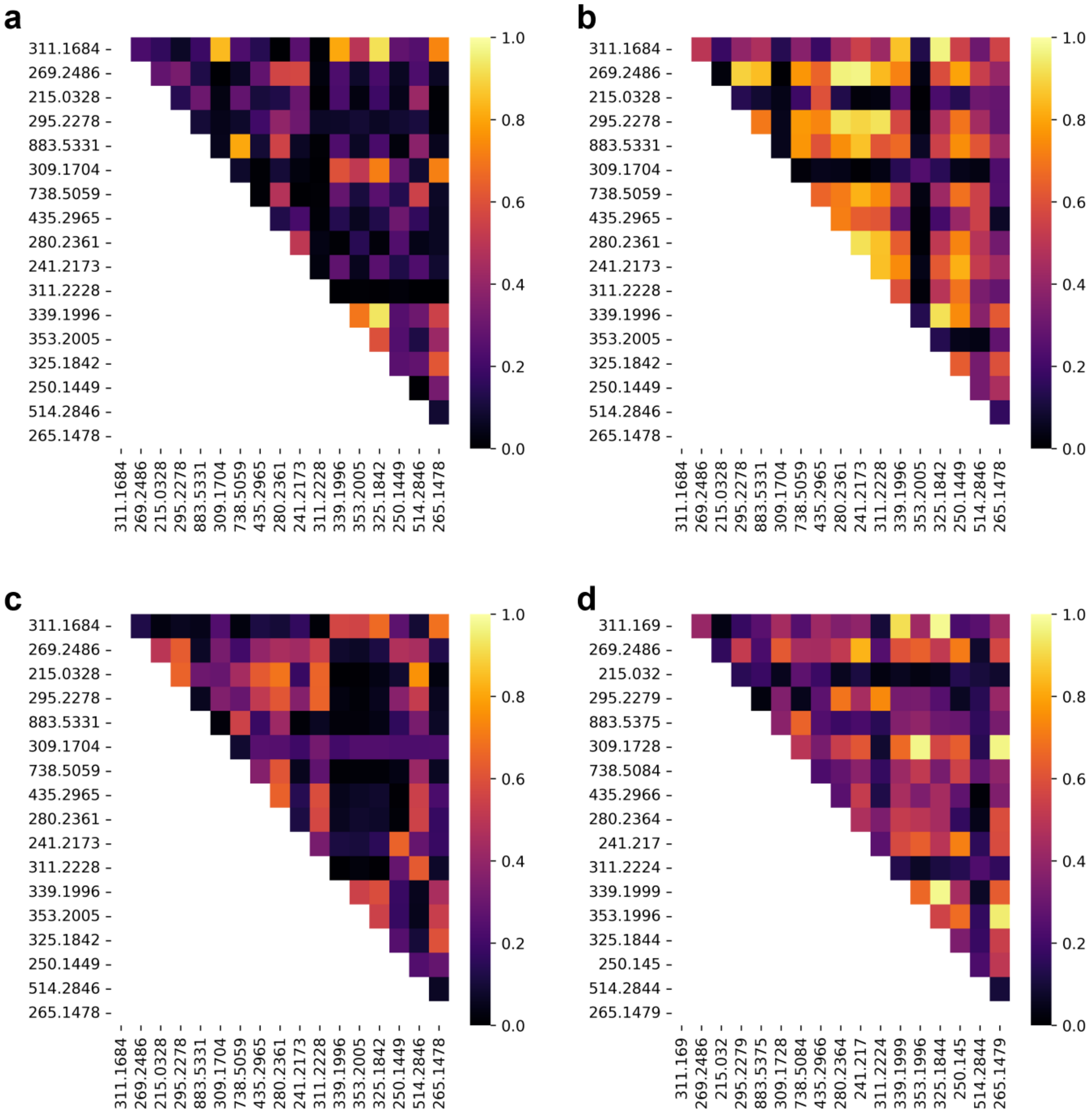

Figure S11: Feature redundancy evaluation for the resolution-adaptive binning approach. Pearson correlation among the 17 candidate features across different datasets, including **(a)** training dataset, **(b)** external dataset, **(c)** fine-needle aspiration glass smear MS imaging dataset, and **(d)** direct infusion MS dataset. Features were retained if their importance scores are higher in a comparison pair or the coefficients of Pearson correlation being less than 0.80.

Figure S12

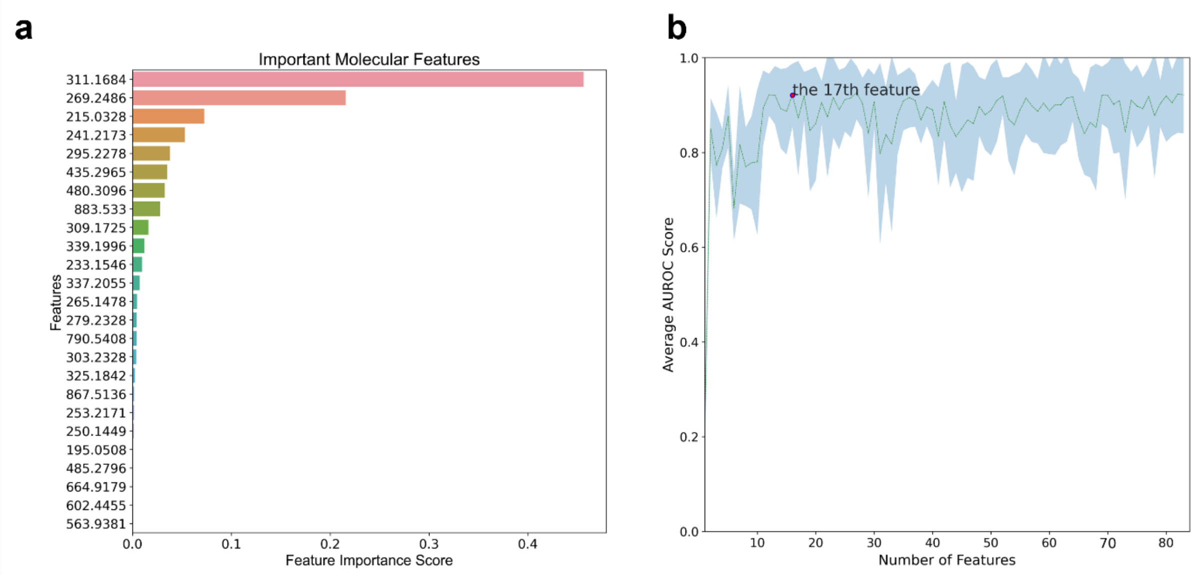

Figure S12: Discriminative feature selection for the dynamic binning approach by 5 ppm. **(a)** Features were ranked based on their feature importance scores derived from the eXtreme Gradient Boosting model. **(b)** Feature selection was performed through model sensitivity analysis. A feature was included if its addition did not lead to 2 consecutive decreases in the average area under the receiver operating characteristics (AUROC) during 5-fold cross-validation.

Figure S13

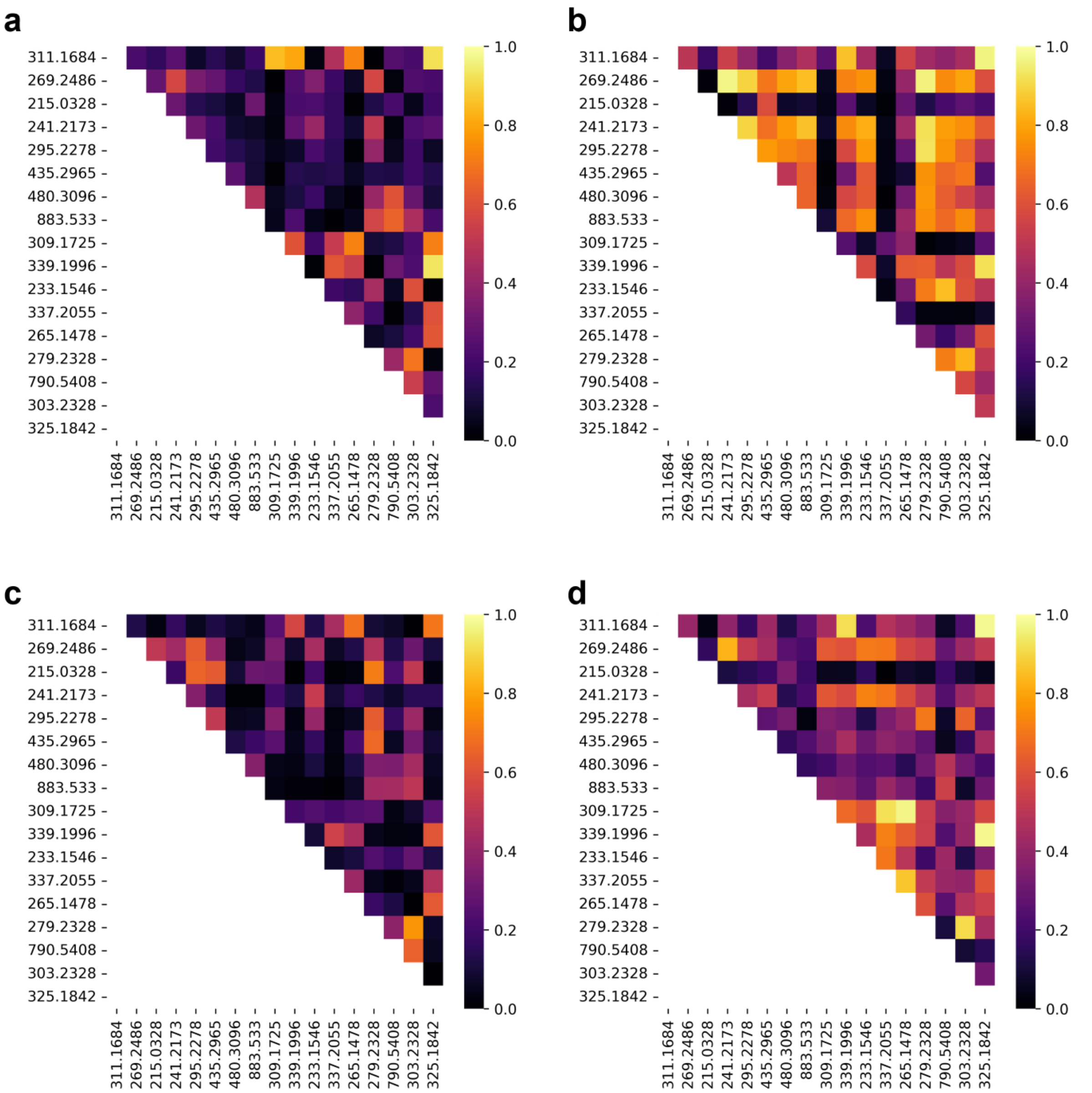

Figure S13: Feature redundancy evaluation for the dynamic binning approach by 5 ppm. Pearson correlation among the 17 candidate features across different datasets, including **(a)** training dataset, **(b)** external dataset, **(c)** fine-needle aspiration glass smear MS imaging dataset, and **(d)** direct infusion MS dataset. Features were retained if their importance scores are higher in a comparison pair or the coefficients of Pearson correlation being less than 0.80.

Figure S14

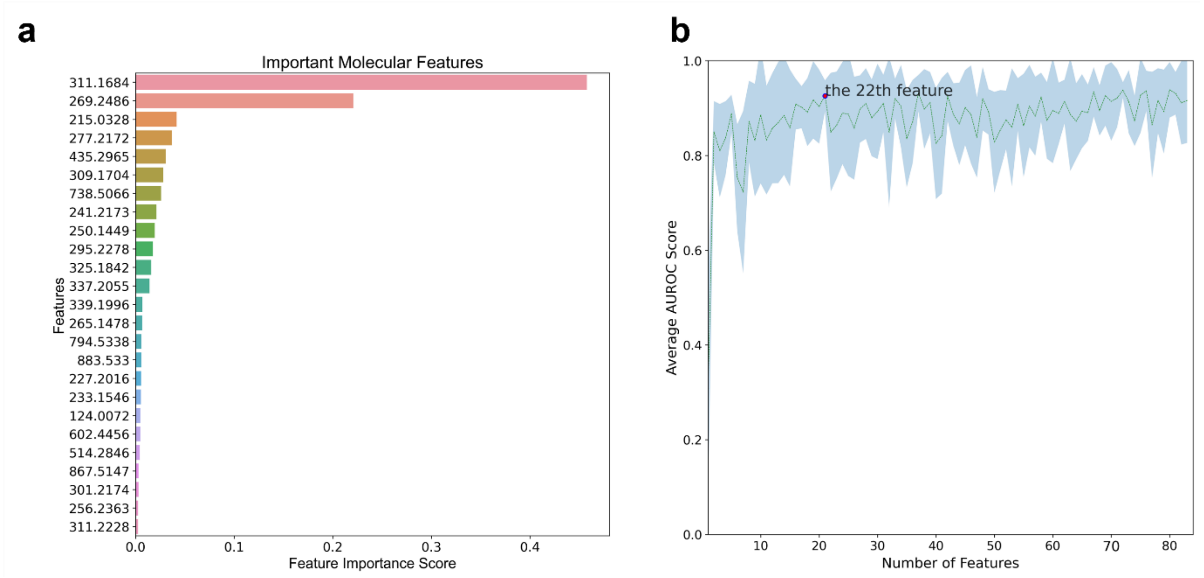

Figure S14: Discriminative feature selection for the dynamic binning approach by 10 ppm. **(a)** Features were ranked based on their feature importance scores derived from the eXtreme Gradient Boosting model. **(b)** Feature selection was performed through model sensitivity analysis. A feature was included if its addition did not lead to 2 consecutive decreases in the average area under the receiver operating characteristics (AUROC) during 5-fold cross-validation.

Figure S15

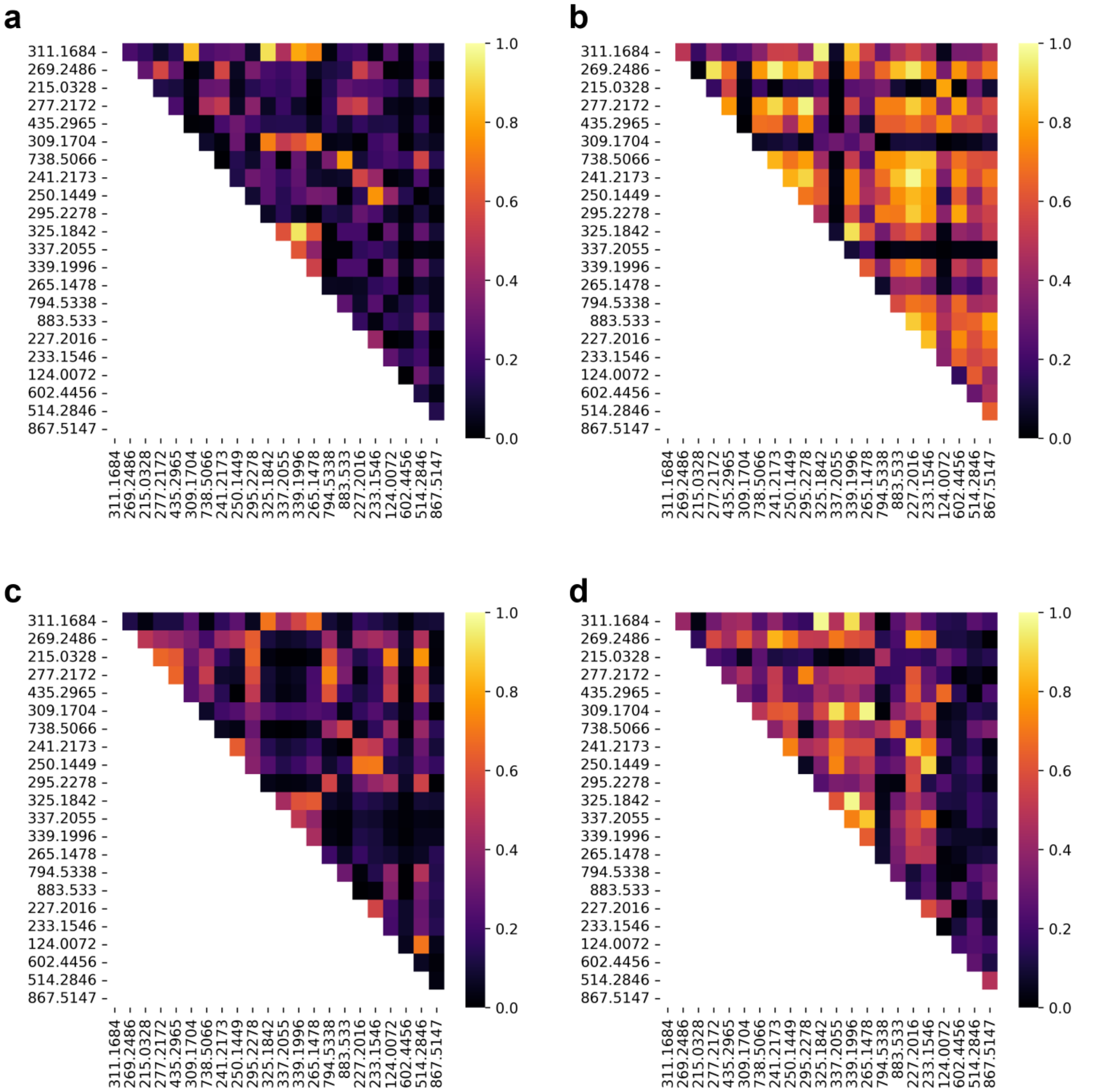

Figure S15: Feature redundancy evaluation for the dynamic binning approach by 10 ppm. Pearson correlation among the 22 candidate features across different datasets, including **(a)** training dataset, **(b)** external dataset, **(c)** fine-needle aspiration glass smear MS imaging dataset, and **(d)** direct infusion MS dataset. Features were retained if their importance scores are higher in a comparison pair or the coefficients of Pearson correlation being less than 0.80.

Figure S16

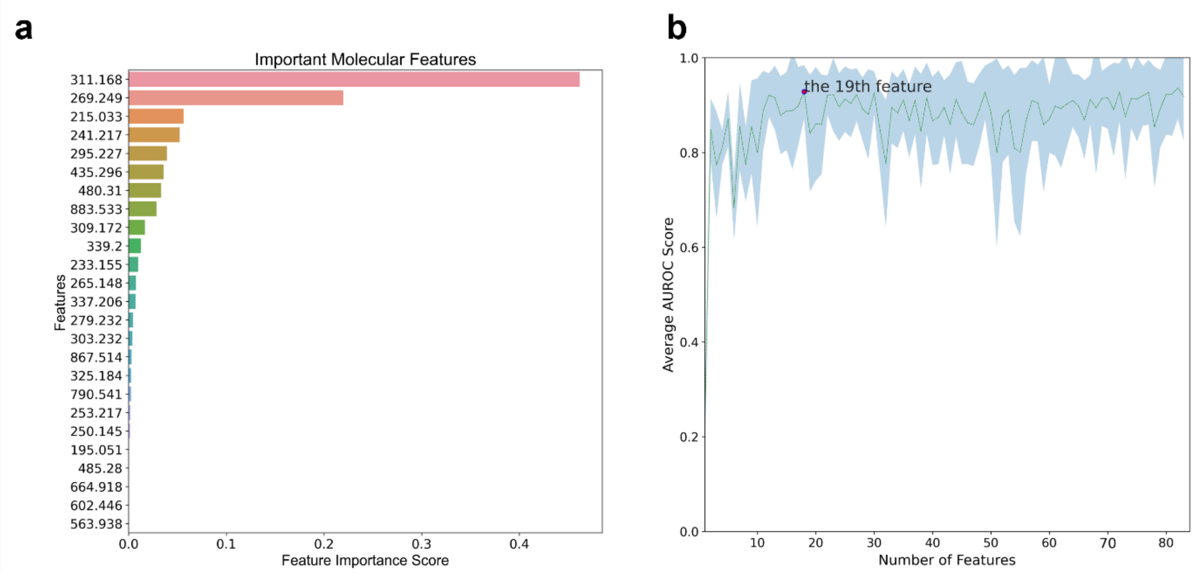

Figure S16: Discriminative feature selection for the constant binning approach by 0.001 Da. (a) Features were ranked based on their feature importance scores derived from the eXtreme Gradient Boosting model. **(b)** Feature selection was performed through model sensitivity analysis. A feature was included if its addition did not lead to 2 consecutive decreases in the average area under the receiver operating characteristics (AUROC) during 5-fold cross-validation.

Figure S17

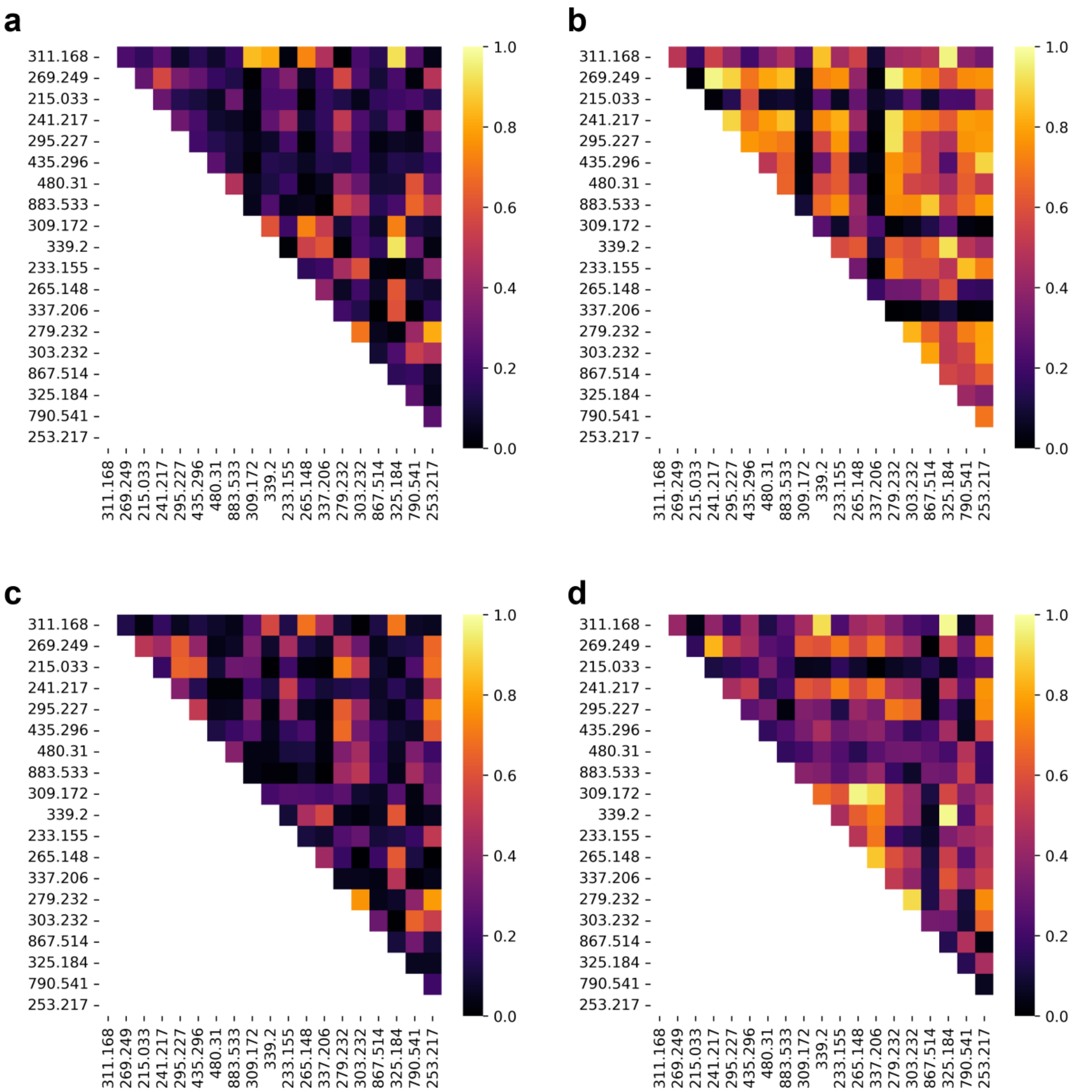

Figure S17: Feature redundancy evaluation for the constant binning approach by 0.001 Da. Pearson correlation among the 19 candidate features across different datasets, including **(a)** training dataset, **(b)** external dataset, **(c)** fine-needle aspiration glass smear MS imaging dataset, and **(d)** direct infusion MS dataset. Features were retained if their importance scores are higher in a comparison pair or the coefficients of Pearson correlation being less than 0.80.

Figure S18

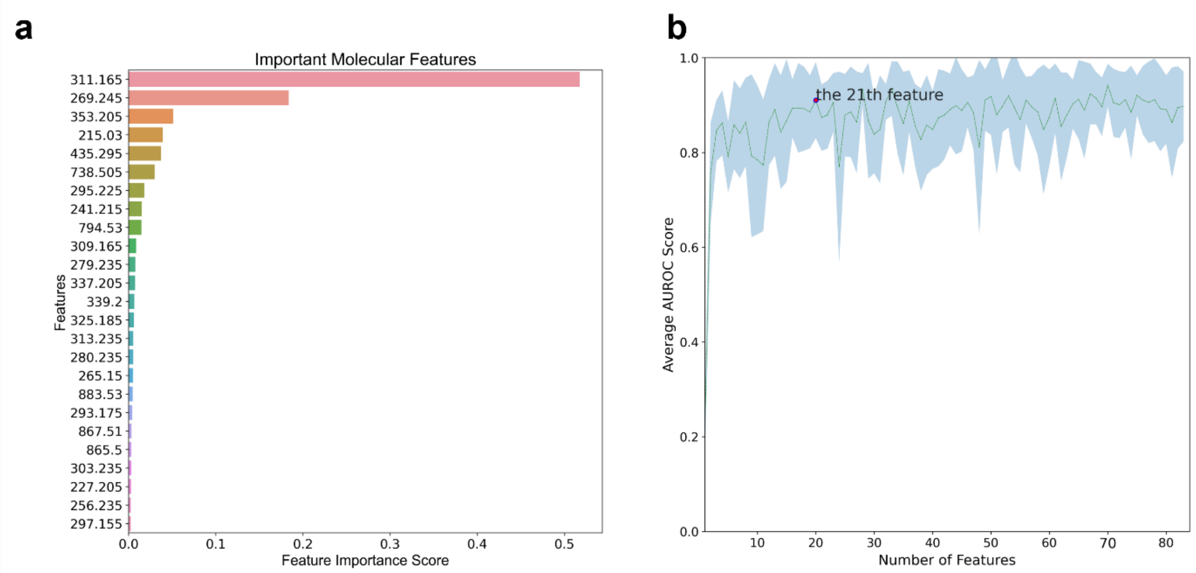

Figure S18: Discriminative feature selection for the constant binning approach by 0.01 Da. (a) Features were ranked based on their feature importance scores derived from the eXtreme Gradient Boosting model. **(b)** Feature selection was performed through model sensitivity analysis. A feature was included if its addition did not lead to 2 consecutive decreases in the average area under the receiver operating characteristics (AUROC) during 5-fold cross-validation.

Figure S19

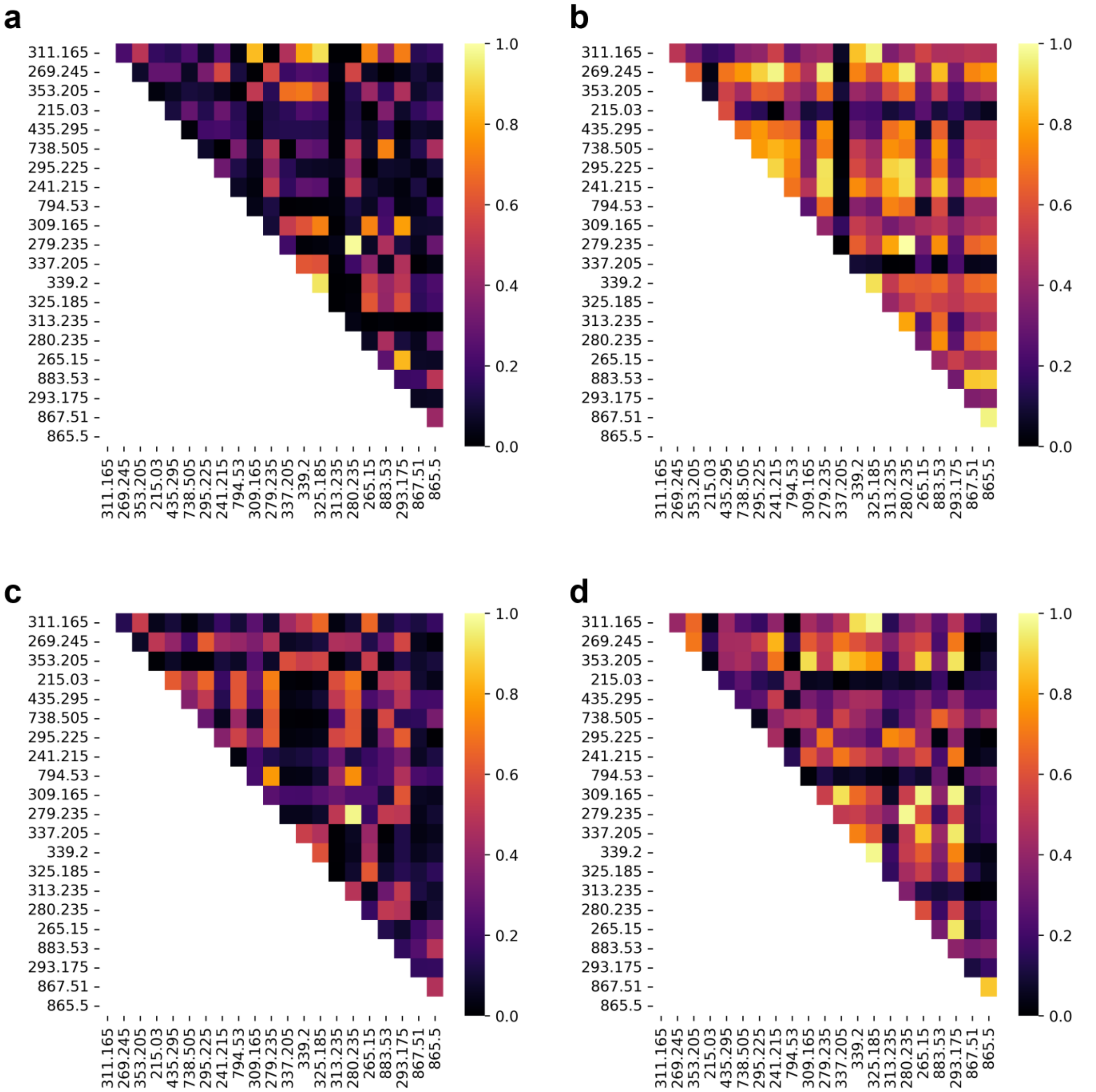

Figure S19: Feature redundancy evaluation for the constant binning approach by 0.01 Da. Pearson correlation among the 21 candidate features across different datasets, including **(a)** training dataset, **(b)** external dataset, **(c)** fine-needle aspiration glass smear MS imaging dataset, and **(d)** direct infusion MS dataset. Features were retained if their importance scores are higher in a comparison pair or the coefficients of Pearson correlation being less than 0.80.

Figure S20

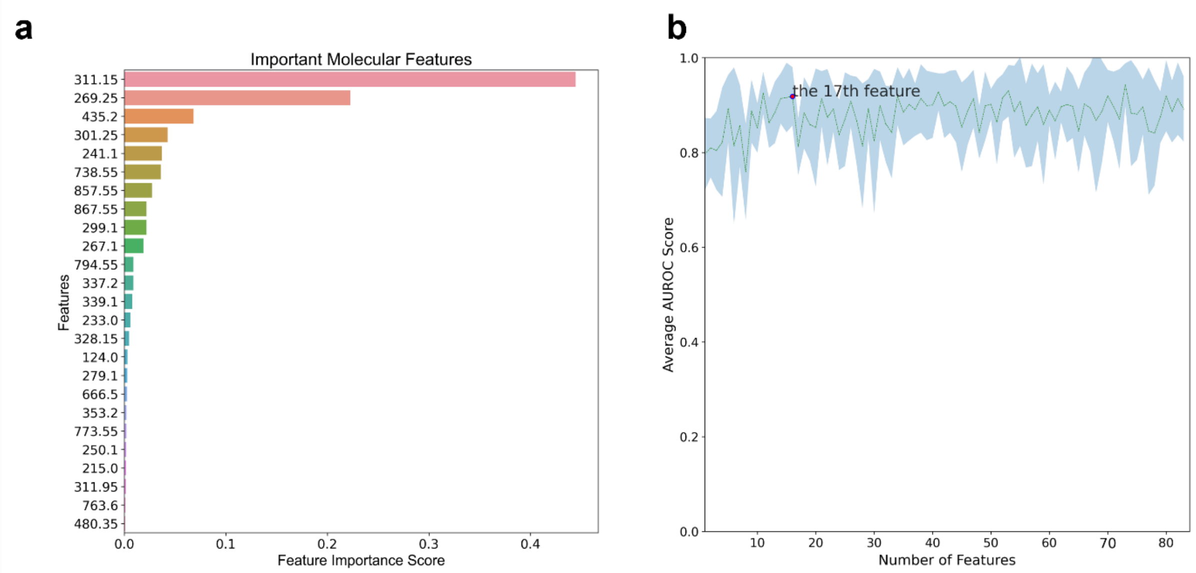

Figure S20: Discriminative feature selection for the constant binning approach by 0.1 Da. (a) Features were ranked based on their feature importance scores derived from the eXtreme Gradient Boosting model. **(b)** Feature selection was performed through model sensitivity analysis. A feature was included if its addition did not lead to 2 consecutive decreases in the average area under the receiver operating characteristics (AUROC) during 5-fold cross-validation.

Figure S21

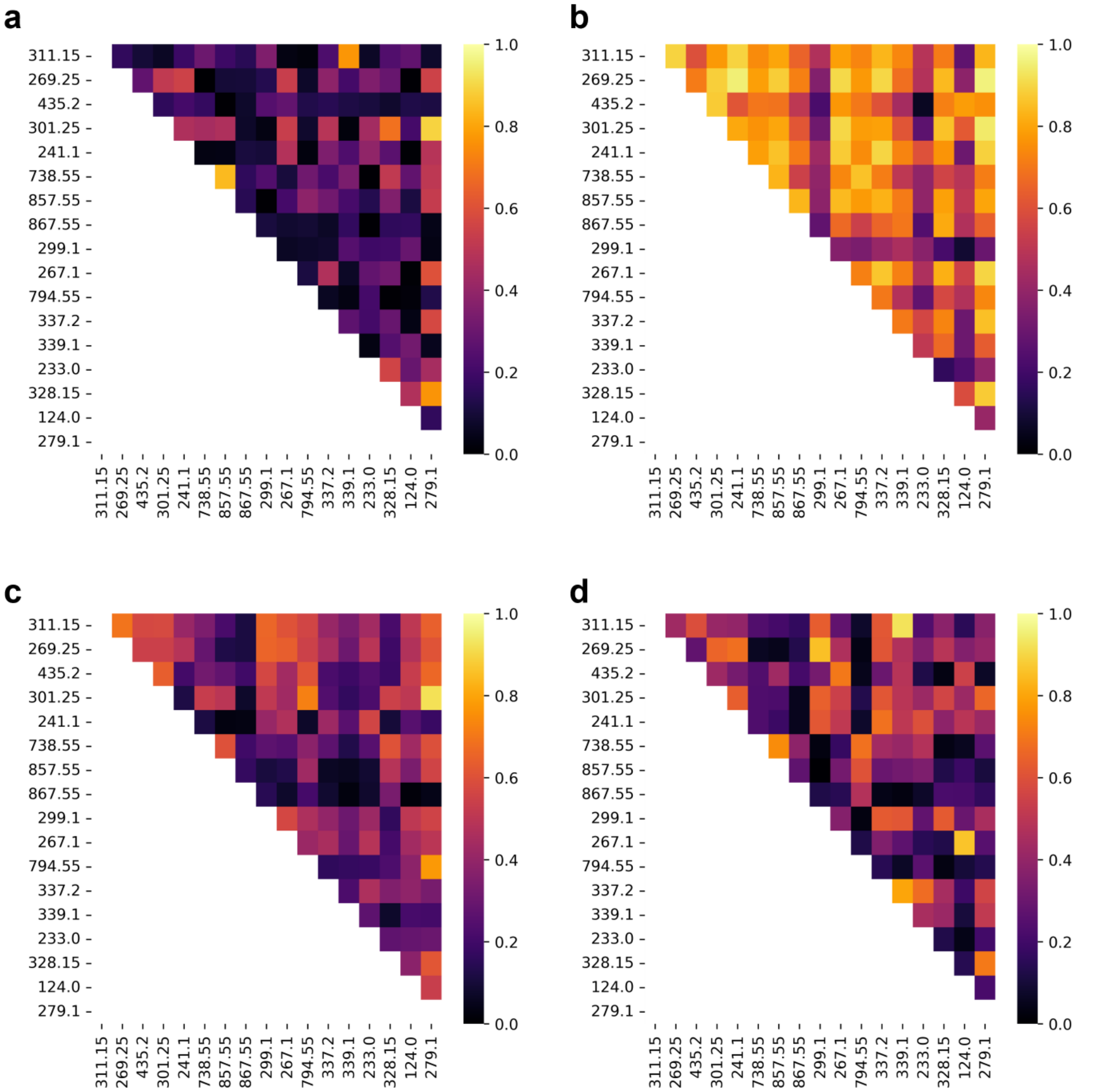

Figure S21: Feature redundancy evaluation for the constant binning approach by 0.1 Da. Pearson correlation among the 17 candidate features across different datasets, including **(a)** training dataset, **(b)** external dataset, **(c)** fine-needle aspiration glass smear MS imaging dataset, and **(d)** direct infusion MS dataset. Features were retained if their importance scores are higher in a comparison pair or the coefficients of Pearson correlation being less than 0.80.

ADDITIONAL TABLES

Table S1: Computational Power and Packages Used.

Workstation	
Edition	Windows 11 Pro
Version	23H2
Installed on	11/10/2023
OS build	22631.4169
Experience	Windows Feature Experience Pack 1000.22700.1034.0
Processor	12th Gen Intel(R) Core(TM) i7-12700K 3.60 GHz
Installed RAM	192 GB (192 GB usable)
System type	64-bit operating system, x64-based processor
Package	
Julia	Pkg, CSV, DataFrames, PyCall, LinearAlgebra, Statistics, ScikitLearn, StatsPlots, Plots
Python	xlwings, pandas, matplotlib, numpy, statistics, numpy, seaborn, sklearn

Table S2: Summary of the Selected Machine Learning Algorithms' Performance and the Comparison of Different Binning Approaches for MS Data Integration.

Datasets	Ingested training[a]		Smear MSI[b]		Direct Infusion[c]		
Metrics	*F*1	MCC	*F*1	MCC	*F*1	MCC	recall
Models (#HCC[d]:#AMC[e])	Resolution-adaptive MS data integration (raMSIn)						
LogReg[f]	0.84	0.68	0.69	0.39			
LinearSVC[g]	0.90	0.80	0.67	0.30			
GB[h]	0.82	0.63	0.70	0.31			
XGB[k]	0.94	0.89	0.67	0.23			
DT[m]	0.92	0.83	0.65	0.07			
RF[p]	0.97	0.94	0.65	0.08			
HardVote (10:10)	0.87	0.74	0.72	0.43	0.66	0.31	0.67
HardVote (6:6)	0.95	0.89	0.56	0.07	0.61	0.24	0.60
HardVote (4:4)	0.68	0.38	0.49	0.17	0.47	0.06	0.42
	Dynamic binning MS data integration: 5 ppm						
HardVote (10:10)	0.88	0.76	0.72	0.42	0.61	0.24	0.60
HardVote (6:6)	0.94	0.87	0.60	0.08	0.61	0.19	0.64
HardVote (4:4)	0.58	0.27	0.46	0.09	0.54	0.23	0.46
	Dynamic binning MS data integration: 10 ppm						
HardVote (10:10)	0.90	0.80	0.71	0.37	0.62	0.23	0.64
HardVote (6:6)	0.93	0.87	0.57	0.12	0.59	0.24	0.55
HardVote (4:4)	0.57	0.06	0.36	-0.14	0.35	-0.15	0.31
	Constant binning MS data integration: 0.001 Da						
HardVote (10:10)	0.86	0.72	0.71	0.41	0.46	0.16	0.37
HardVote (6:6)	0.93	0.86	0.59	0.18	0.38	-0.03	0.31
HardVote (4:4)	0.40	-0.08	0.44	-0.03	0.44	-0.01	0.39
	Constant binning MS data integration: 0.01 Da						
HardVote (10:10)	0.87	0.73	0.66	0.26	0.63	0.27	0.61
HardVote (6:6)	0.92	0.86	0.54	0.09	0.55	0.23	0.47
HardVote (4:4)	0.74	0.48	0.53	0	0.49	-0.04	0.49
	Constant binning MS data integration: 0.1 Da						
HardVote (10:10)	0.85	0.69	0.72	0.41	0.50	-0.07	0.53
HardVote (6:6)	0.92	0.85	0.56	0.25	0.33	-0.20	0.30
HardVote (4:4)	0.54	0.40	0.55	0.31	0.35	-0.05	0.28

[a] Ingested training dataset was prepared by integrating 2 independent batches of desorption electrospray ionization mass spectrometry imaging (DESI-MSI) data of cryosection tissue samples (training batch, *N*=12, *n*=90,960; external batch, *N*=8, *n*=6,075; total: 20 animal subjects and 97,035 MS1 pixels of shotgun analysis).

[b] A testing dataset that was prepared from an independent batch of DESI-MSI shotgun data of glass smears. Fine-needle aspirate samples were collected at the loci outside tumor regions (*N*=6, *n*=88,701).

[c] Another testing dataset that was prepared from an independent batch of direct infusion MS1 data of tissue extract. Fine-needle aspirate samples were collected at the loci outside tumor regions (*N*=10, *n*=6,057).

[d] Number of animal subjects were belonging to the hepatocellular carcinoma (HCC) model.

[e] Number of animal subjects were belonging to the age-matched control of the HCC model.

[f] Logistic Regression.

[g] Linear Support Vector Classifier.

[h] Gradient Boosting.

[k] eXtreme Gradient Boosting.

[m] Decision Tree.

[p] Random Fores.

Table S3: Summary of 10 Annotated Discriminative Metabolites that Contributed to Hepatocellular Carcinoma Prediction.

m/z (-ve mode)	Compound SubClass	Annotation	MSn	Grade/ Level	Adduct	Formula	Delta PPM	RT. error	Source	Fold Change
311.1684	Linoleic acids and derivatives	13(S)-HpODE	2	1	M-H	C18 H31 O4	2E-01	0.41	HMDB 0003871	1.40
		9(S)-HpODE	2	1	M-H	C18 H31 O4	2E-01	0.41	HMDB 0062434	
269.2486	Straight chain fatty acids	FA 17:0	2	C/2B	M-H	C17 H33 O2	-5E-01		LipidSearch	0.30
215.0328	Fatty acids and conjugates	Undecane-dioic acid	2	1	M-H	C11 H20 O4	2E-02	1.65	HMDB 0000888	1.72
295.2278	Unsaturated fatty acids	FA(18:1)+ OX:(s)	2	C/2B	M-H	C18 H32 O3	3E-01		LipidSearch	0.10
883.5331	diacyl	PI(18:1_20:4)	2	A/2B	M-H	C47 H81 O13 P1	-9E-01		LipidSearch	1.25
		PI(18:0_20:5)	2	A/2B	M-H	C47 H81 O13 P1	-9E-01		LipidSearch	
309.1704	Fatty acids and conjugates	FA 17:4;O3	1	3	M-H	C17 H26 O5	-1E+00		LMFA 02030076<br>LMFA 01050151	0.61
738.5059	diacyl	PE(16:0_20:4)	2	A/2B	M-H	C41 H74 N1 O8 P1	5E-02		LipidSearch	1.44
		PE(18:2_18:2)	2	A/2B	M-H	C41 H74 N1 O8 P1	5E-02		LipidSearch	
435.2965	Monoradyl-glycerols	2,3-Dihydroxy-propyl octanoate	1	3	2M-H	C11 H22 O4	1E+00		HMDB 0254850	0.64
250.1449	Diacyl-glycero-phospho-serines	PS 34:5	1	3	M-3H	C40 H68 N1 O10 P1	-2E+00		LMGP 03010922<br>LMGP 03010131<br>LMGP 03010899<br>LMGP 03010432<br>LMGP 03010623<br>LMGP 03010650	1.17
514.2846	Bile acids, alcohols and derivatives	Tauro-alpha-muricholic acid	2	1	M-H	C26 H45 N1 O7 S1	2E-01	5.32	HMDB 0258742	4.54
		Tauro-b-muricholic acid	2	1	M-H	C26 H45 N1 O7 S1	2E-01	11.92	HMDB 0000932	
		Tauro-cholic acid	2	1	M-H	C26 H45 N1 O7 S1	6E-01	15.75	HMDB 0000036	

Table S4: Sources of the High-Resolution MS Data.

MS Platform	Study identifier	Sample introduction method	Data file name	Sample type	Mass resolution	URL
			**Publicly Available (Q-)Orbitrap Data**			
Orbitrap_Elite	MTBLS614	C18-LC/ pESI-MS data	"OBOBWTLiver28.raw"	Mouse liver	60k @200 *m/z*	https://www.ebi.ac.uk/metabolights/editor/MTBLS614/descriptors
Q_Exactive_Plus	MTBLS2397	Zic-pHilic-LC/ pESI MS data	"IN-07032017-pHILIC-Spain-Liver-858.raw"	Mouse liver	35k @200 *m/z*	https://www.ebi.ac.uk/metabolights/editor/MTBLS2397/descriptors
LTQ-Orbitrap	MTBLS61	C18-LC/ nESI-MS data	"F_KO_7.RAW"	Mouse plasma	30k @200 *m/z*	https://www.ebi.ac.uk/metabolights/editor/MTBLS61/descriptors
	MTBLS281	RPLC/ pESI-MS data	"mtab_sponge_ft_16.RAW"	Seawater	97k @400 *m/z*	https://www.ebi.ac.uk/metabolights/editor/MTBLS281/descriptors
FT-ICR-MS	rat_eye_rms	-ve MALDI MSI data	"rat_eye_rms"	Rat eye	100k @400 *m/z*	https://metaspace2020.org/datasets?q=rat_eye_rms
ToF-MS	brain-9aa-neg-40um	-ve MALDI MSI data	"brain-9aa-neg-40um"	Mouse brain	25k @200 *m/*	https://metaspace2020.org/dataset/2023-08-25_17h02m43s
			**In-house (Q-)Orbitrap Data**			
Orbitrap_Fusion	/	C18-LC/ pESI-MS data	/	Mouse serum	120k @200 *m/z*	can provide upon request
Q Exactive	/	T3-LC/ nESI -MS data	/	Mouse liver	70k @200 *m/z*	can provide upon request
Q_Exactive_Focus	/	T3-LC/ nESI-MS data	/	Mouse liver	70k @200 *m/z*	can provide upon request
Orbitrap_Exploris120	/	nDESI MSI data	/	mouse liver section	60k @200 *m/z*	can provide upon request
